# Magnetic activation of spherical nucleic acids enables the remote control of synthetic cells

**DOI:** 10.1038/s41557-025-01909-6

**Published:** 2025-09-02

**Authors:** Ellen Parkes, Assala Al Samad, Giacomo Mazzotti, Charlie Newell, Brian Ng, Amy Radford, Michael J. Booth

**Affiliations:** 1 Department of Chemistry, University of Oxford, Oxford, UK; 2 Department of Chemistry, University College London, London, UK

**Keywords:** Synthetic biology, Nanoparticles, Nucleic acids

## Abstract

The flexible and modular design of synthetic cells, comprising lipid vesicles capable of imitating the structure and function of living cells, facilitates their application as drug delivery devices. The ability to control the synthesis of biomolecules within synthetic cells using a tissue-penetrating stimulus opens up additional levels of functionality that has the potential to improve biological potency and circumvent drug leakage from preloaded vesicles. To this end, we have designed spherical nucleic acids comprising DNA promoter sequences decorating magnetic nanoparticle cores. These spherical nucleic acids allowed us to harness the heat dissipated from magnetic hyperthermia (a clinically approved anticancer therapy) to regulate cell-free protein synthesis and release cargo on demand. Furthermore, this magnetic regulation of biosynthesis was achieved using clinically tolerable magnetic field strengths and frequencies. We then deployed an opaque blocking material that is impenetrable by current activation methods to highlight the potential of this technology for targeting and controlling the in situ synthesis of biomolecules using tissue-penetrating magnetic fields deep within the body.

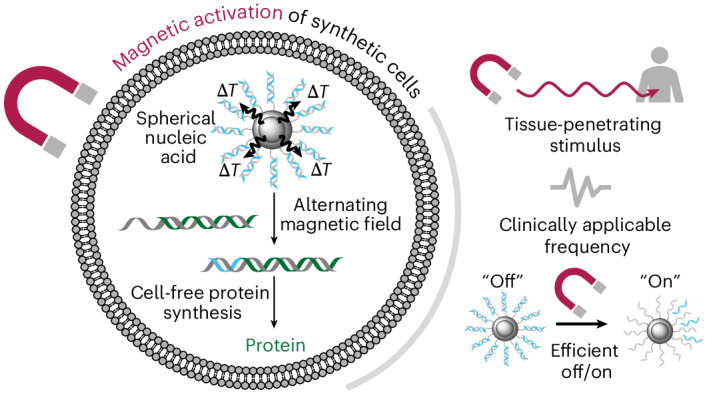

## Abstract (web summary)

The programmability of synthetic cells, comprising lipid vesicles that are capable of imitating the structure and function of living cells, facilitates their application as drug delivery devices. Now, magnetic hyperthermia has been used to control the on-demand synthesis and release of biomolecules from within synthetic cells.

## Main

In recent years the complexity of reprogramming living cells has driven research efforts into constructing synthetic mimics using modular parts. The biomimicry of enzymatic reactions and stimuli-responses has been achieved in synthetic cells; non-living compartments capable of imitating the behaviour, function and structure of living cells^1,2^. The majority of synthetic cells comprise a cell-free protein synthesis (CFPS) system bounded by a lipid membrane, programmed according to DNA templates that are specific to the desired biological activity^3^. Notably, synthetic cells can accommodate non-biological components to attain new functionalities that are not accessible to living cells, which substantially increases their technological value by opening up applications as novel drug delivery systems and bioreactors^2,4^. Indeed, synthetic cells loaded with CFPS systems have been administered in vivo, proving non-immunogenic and stable^5–7^.

A prerequisite for the application of synthetic cells to biology and medicine is the ability to control their activity, reducing off-target effects and improving therapeutic potential^8^. Modulating CFPS at the DNA or mRNA level has been achieved using small-molecule-sensitive transcription factors and riboswitches, although approaches of these kinds are limited in synthetic cells due to the difficulty of small molecules traversing the membrane^9–11^. By using an external stimulus, remote regulation of internal CFPS can be achieved in synthetic cells with a level of spatial precision not accessible to small-molecule techniques^8,12^. The majority of research in this area currently focuses on using ultraviolet (UV) light, which poses cytotoxicity issues and has limited ability to penetrate bodily tissue (by <1 mm), hindering its use in therapeutic applications^8^. Beyond this, temperature has been explored as an external stimulus to control CFPS and small-molecule release with RNA thermometers, albeit at temperatures outside of the biologically useful range^13^. An ideal external stimulus for controlling CFPS within synthetic cells is alternating magnetic fields (AMFs), which are deeply tissue penetrating (by >10 cm) and biologically benign^14,15^. Superparamagnetic nanoparticles dissipate thermal energy to the surrounding medium as a result of Brownian and Néel fluctuations in the presence of an AMF^16^. Brownian relaxation is described as the physical rotation of the particle, whereas Néel relaxation is described as the rotation of the magnetic moment within the particle. If the applied AMF is strong enough to rotate the particle itself or to displace the magnetic moment from the preferred orientation, then relaxation back to equilibrium releases thermal energy to the surroundings^17^. This localized heating, termed magnetic hyperthermia, is clinically approved to ablate malignant tumours by exploiting the differing heat tolerance between tumour and healthy cells, whereby healthy cells can survive above physiological temperature for longer^18^. Superparamagnetic iron oxide nanoparticles (SPIONs) (typically magnetite and/or maghaemite) have been extensively researched due to their inherent biocompatibility, forming the basis of clinically approved and globally marketed medicines as iron supplements and contrast agents for magnetic resonance imaging (MRI)^19,20^. Superparamagnetic materials are of particular interest for biomedical applications because they do not exhibit coercivity or remanent magnetization, thereby avoiding issues arising from magnetic aggregation in blood vessels and imparting a high magnetic susceptibility^21^. Magnetic hyperthermia has also been investigated for rupturing lipid bilayers, but it demands high frequencies that are neither clinically tolerable nor compatible with current medical AMF generators^22–25^. Additionally, the disruption of bilayers limits their potential use with synthetic cells and CFPS.

We have designed a magnetically controllable CFPS system for use in synthetic cells by taking inspiration from spherical nucleic acids (SNAs), a delivery platform comprising nucleic acids that are radially arranged around a nanoparticle core^26^. We have synthesized SNAs with magnetic cores to harness the heat dissipated from magnetic hyperthermia and release a T7 promoter sequence, enabling the recovery of an otherwise inactive DNA template and the subsequent spatiotemporal control of its activity. This mechanism has previously been applied to measure the surface temperature of the nanoparticles and to release DNA in vivo, but required high electromagnetic frequencies that are not clinically compatible^14,27,28^. The only clinically available AMF system operates at a frequency of 100 kHz, chosen to minimize eddy currents within the body and reduce patient discomfort^25^. In light of this, there is a need to develop systems that operate within clinically relevant magnetic field frequencies to aid medical translation. In addition, these previous studies did not control the activity and downstream function of the attached DNA. Through nanoparticle engineering and the development of our SNA purification method, we were able to activate gene-expressing synthetic cells using clinically tolerable and Food and Drug Administration/European Medicines Agency-approved frequencies (100 kHz). This technology paves the way for the application of synthetic cells as a widespread therapeutic modality and realizes their potential as on-demand drug delivery devices with enhanced tissue penetration.

## Magnetic nanoparticle engineering

**Fig. 1:**
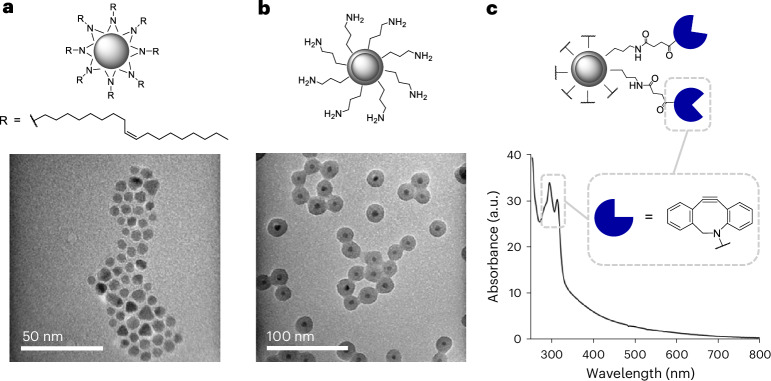
Silica-encapsulated iron oxide nanoparticle construction and their modification with the DBCO click handle. **a**, Schematic and TEM image of oleylamine-capped IONPs synthesized by thermal decomposition. **b**, Schematic and TEM image of NH_2_-modified IONPs@SiO_2_ synthesized by reverse microemulsion. **c**, Schematic and UV–vis spectrum of DBCO-modified IONPs@SiO_2_ synthesized by NHS ester-coupling, highlighting the characteristic DBCO absorption. Source data

To create our magnetically activated SNAs, we designed the central scaffold to consist of silica-encapsulated iron oxide nanoparticles (IONPs@SiO_2_) (Fig. 1). The IONP core was maintained in the superparamagnetic regime (<100 nm) to utilize the heat generated by Brownian and Néel relaxations in an AMF for downstream heat-induced nucleic acid release. The subsequent encapsulation in silica allowed for the grafting of primary amines, in-turn allowing for easy surface modification through *N*-hydroxysuccinimide (NHS) ester chemistry. We synthesized IONPs by thermally decomposing iron(III) acetylacetonate in the presence of oleylamine and benzyl ether^29,30^. The hydrophobic oleylamine-capped IONPs were of uniform shape and narrow size distribution, as confirmed by transmission electron microscopy (TEM) with a diameter of *D*_TEM_ = 6.4 ± 1.0 nm (Fig. 1a). Further structural information was obtained by X-ray diffraction (XRD), which indicated that the oleylamine-capped IONPs were crystalline and phase pure with the position and relative intensity of the diffraction peaks matching that of magnetite (RUFF ID: R061111) (Supplementary Fig. 1a)^31^. The crystallite size calculated from the Scherrer equation at *D*_XRD_ = 6.2 nm was comparable to that determined by statistical analysis of the TEM images, indicating that each particle was a single crystal^32^. Dynamic light scattering (DLS) was utilized to investigate the hydrodynamic size of the nanoparticles, which proved to be larger than the crystallite size at *D*_DLS_ = 8.4 nm, taking into account the fluid interactions and adsorbed surfactant (Table 1). The hysteresis curve measured at 300 K determined that the oleylamine-capped IONPs were superparamagnetic at room temperature with zero coercivity, in turn confirming that the net magnetization in the absence of an external field was zero (Supplementary Fig. 1b). Their saturation magnetization plateaued at 56.0 emu g_Fe_^−1^ while their magnetic susceptibility (*χ*_max_) equalled 0.049 emu g^−1^ Oe^−1^ at 68.5 Oe; the former property influences the thermal energy dissipated in an AMF, with high saturation magnetism values resulting in greater inductive heating^33^.

**Table 1:** Nanoparticle characterization

Nanoparticle	Hydrodynamic diameter (nm)	PDI	*ζ*-potential (mV)
Oleylamine-capped IONPs	8.4	0.43	NA
NH_2_-modified IONPs@SiO_2_	36.7	0.38	17.8
DBCO-modified IONPs@SiO_2_	142.0	0.20	−13.9
dsDNA T7-modified IONPs@SiO_2_ (SNA)	125.3	0.38	−36.6

Summary of the hydrodynamic diameter (analysis in number), polydispersity index (PDI) and *ζ*-potential, as determined by DLS with the mean calculated from *n* = 3 repeat measurements. NA, not available.

To obtain biocompatible particles with a readily modifiable surface chemistry, we coated the IONPs in silica (SiO_2_) (Fig. 1b). The reverse microemulsion route was chosen for enhanced morphological control and reduced yields of by-product SiO_2_ with no IONP core^34^. Briefly, the oleylamine-capped IONPs underwent a ligand exchange with the surfactant IGEPAL-*co*-520 in cyclohexane, prior to the addition of concentrated aqueous ammonium hydroxide that nucleated the tetraethylorthosilicate precursor within the aqueous domains, forming SiO_2_ shells around single IONP cores. In the final step (3-aminopropyl)triethoxysilane was injected to modify the surface of the IONPs@SiO_2_ with reactive amine (NH_2_) groups. The NH_2_-modified IONPs@SiO_2_ were characterized by TEM, revealing monodisperse particles with a diameter of *D*_TEM_ = 20.9 ± 1.8 nm and no observed by-product SiO_2_ (Fig. 1b). DLS was utilized to investigate the hydrodynamic size after the SiO_2_ encapsulation, which increased to *D*_DLS_ = 36.7 nm. The zeta (*ζ*) potential further indicated the addition of protonatable NH_2_ groups with a measured charge of *ζ* = +17.8 mV (Table 1). A copper-free click handle was installed on the surface of the NH_2_-modified IONPs@SiO_2_ by coupling to an NHS ester-modified dibenzocyclooctyne (DBCO). The success of the reaction was tracked by ultraviolet–visible (UV–vis) spectroscopy and the absorbance of DBCO at its excitation maximum wavelength of 309 nm (Fig. 1c). The Beer–Lambert law was used to decipher the concentration of DBCO at a molar extinction coefficient of *ε* = 12,000 M^−1^ cm^−1^ (309 nm) (Supplementary Equations (5) and (6))^35^. The particle concentration was determined by approximating individual nanoparticles as spherical in shape and relating the volume and area extrapolated from TEM analysis to known densities of magnetite and silica (Supplementary Equations (1)–(4))^36–38^. The resulting number of DBCO molecules decorating each core–shell nanoparticle averaged 3,669. DLS measurements inferred the successful modification by DBCO with an increase in hydrodynamic diameter from *D*_DLS_ = 36.7 nm for NH_2_-modified IONPs@SiO_2_ compared with *D*_DLS_ = 142.0 nm for DBCO-modified IONPs@SiO_2_ and further a shift in surface charge from *ζ* = +17.8 mV to *ζ* = −13.9 mV (Table 1), consistent with the change in surface chemistry.

## Spherical nucleic acid synthesis

**Fig. 2:**
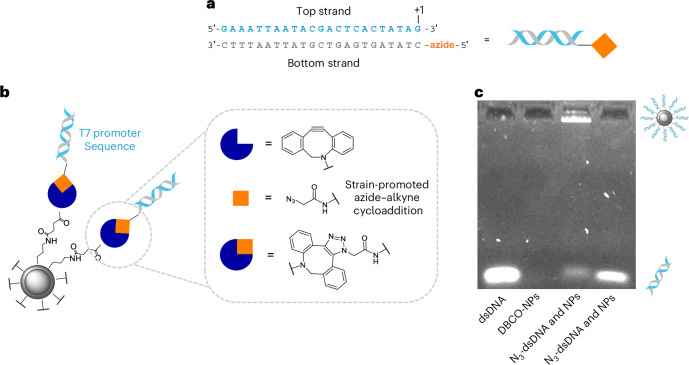
SNA synthesis and purification. **a**, Schematic showing the top and bottom (azide-modified) T7 promoter strands. The transcription initiation site (+1) is marked. **b**, Schematic detailing the covalent attachment of the T7 promoter strands to the surface of the nanoparticle to form the magnetically activated SNAs. The azide-modified, double-stranded T7 promoter enabled copper-free click chemistry to the DBCO-modified nanoparticles. **c**, Agarose gel showing the SNA purification method to remove electrostatically bound DNA from the surface of the nanoparticles using an electric current. The covalently constructed SNA (with the azide-modified DNA) did not travel through the gel and was then extracted through brief sonication of the excised well in water. DNA that was not covalently attached to the surface of the nanoparticles was pulled off by applying an electric current to the agarose gel and can be seen to travel through the gel at the same speed as the free DNA and amine-only modified dsDNA incubated with the nanoparticles. Source data

To form the SNAs, we used copper-free, strain-promoted azide–alkyne cycloaddition to conjugate azide-modified double-stranded (ds) DNA, comprising the T7 promoter strand, to the DBCO-modified IONPs@SiO_2_ (Fig. 2a,b). In this system, the ‘bottom’ T7 promoter strand was covalently attached to the nanoparticles with the ‘top’ T7 promoter strand hybridized to the bottom strand. Tethering the dsDNA to the IONPs@SiO_2_ was important to maximize the localized heating experienced by the dsDNA on account of the heat generated by the IONPs and to influence the extent of T7 promoter release for downstream application. The bottom strand of the T7 promoter was sourced with a 5ʹ-azide group to enable conjugation to the surface of the nanoparticles. This azide-modified bottom T7 promoter strand was annealed to an unmodified top T7 promoter strand and analysed by polyacrylamide gel electrophoresis (PAGE) (Supplementary Fig. 2). Only one band associated with the sole dsDNA product was observed and so no purification of the annealed dsDNA product was required. The SNA synthesis was carried out by freezing the azide-modified dsDNA in the presence of DBCO-modified IONPs@SiO_2_ and low concentrations of salt^39^. The freeze-directed construction of SNAs takes advantage of ice crystals gradually forming, concentrating the reagents into ‘micro-pockets’ and alleviating steric and electrostatic hindrances, thereby encouraging SNA formation^39,40^. We identified high concentrations of DNA electrostatically bound to the nanoparticles (alongside the formation of covalently bound DNA) with conventional SNA synthesis methods. This was detrimental to controlling the activity of the attached DNA because the electrostatically bound DNA leached off in biological conditions (Supplementary Fig. 3a). To mediate this, we developed a purification method to produce SNAs decorated solely with covalently bound DNA. Our purification involved pulling electrostatically bound DNA from the surface of the nanoparticle using an electric current that was applied through an agarose gel; the covalently constructed SNA did not travel through the gel and was then extracted through brief sonication of the excised well in water (Fig. 2c). We found that this purification step was essential to rendering the SNA inactive in the absence of the AMF, whereby a leaky ‘off’ state was observed before agarose purification with the expression of RNA, determined by the generation of a fluorescent RNA–fluorophore complex, averaging six times higher than that observed after purification and in the absence of the magnetic field (Supplementary Fig. 3a). This is probably due to the electrostatically bound DNA leaching off the surface of the SNAs and now being free in solution to participate in the downstream function. The concentration of electrostatically bound DNA on the surface of the SNAs was determined before and after agarose purification by comparing the amount of dsDNA pulled off the SNAs, travelling through the agarose gel on account of the applied electric current, to a calibration curve of known concentration. The concentration of electrostatically bound DNA on the surface of the SNAs decreased by a factor of 10 after agarose purification (Supplementary Fig. 4a,b).

The resulting magnetically activated SNAs remained colloidally stable as determined by the highly negative *ζ*-potential induced by the phosphate DNA backbone, decreasing to *ζ* = −36.6 mV. The decrease in hydrodynamic diameter to *D*_DLS_ = 125.3 nm upon SNA formation is indicative of the newly formed and monodisperse colloid, stabilized by DNA-to-DNA electrostatic repulsion (Table 1). The loading of the T7 promoter was determined by denaturing the dsDNA attached to the IONPs@SiO_2_ at 95 °C for 5 min in urea-containing dye and comparing the amount of released DNA to a calibration curve of known concentration on an agarose gel (Supplementary Fig. 5a,b; see Supplementary Information for details). The calculated concentration of attached T7 promoter strands was related to the previously calculated nanoparticle concentration to give an estimated loading of 18 dsDNA strands per nanoparticle, which is in line with literature values for SNAs comprising dsDNA^39^.

## Magnetic activation of cell-free protein synthesis

**Fig. 3:**
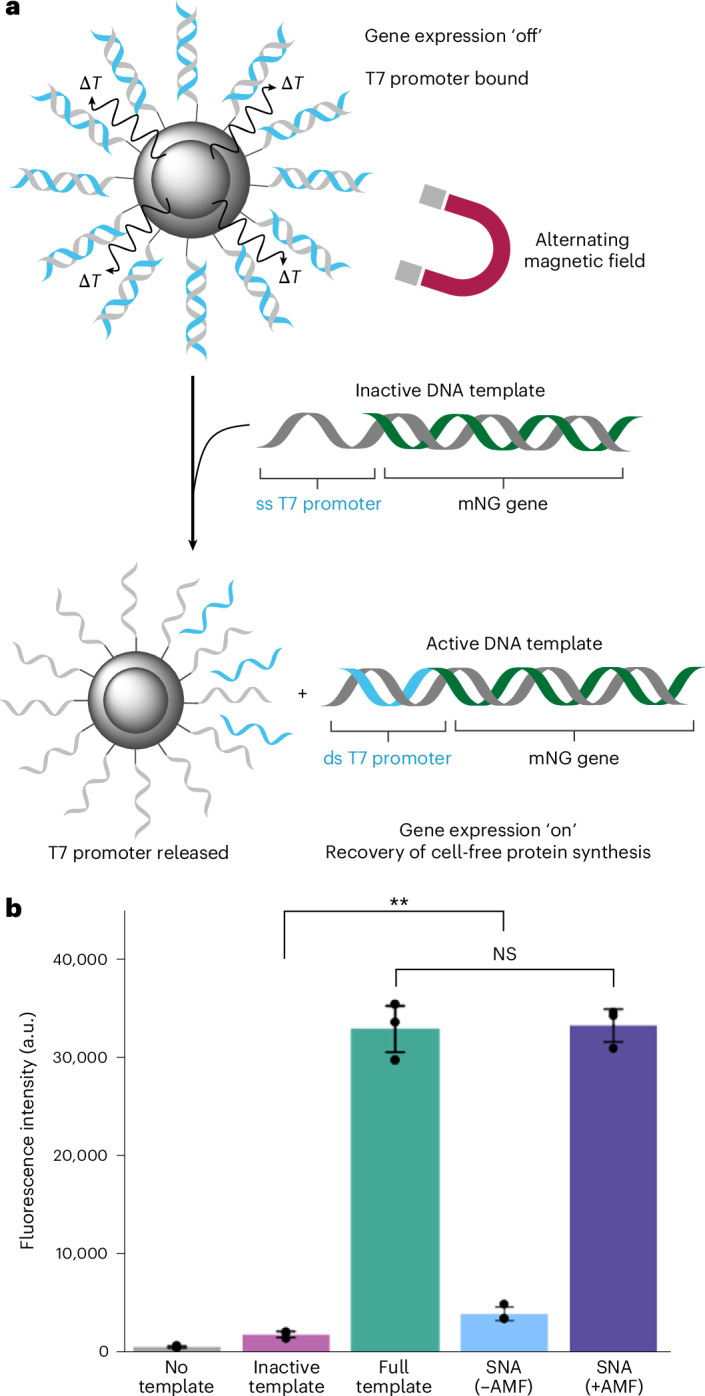
Controlling cell-free protein synthesis with an alternating magnetic field. **a**, Schematic detailing the release of the T7 promoter top sequence from the SNA surface as a result of inductive heating and exposure to an AMF, and the subsequent hybridization of the promoter sequence to the single-stranded T7 promoter of the inactive template, restoring the dsDNA T7 promoter and activating expression of the encoded fluorescent mNG protein. Note: promoter (23 bp) and gene (∼700 bp) length are not to scale. *T*, temperature. **b**, Cell-free protein synthesis of mNG in the presence of magnetically activated SNAs, with and without exposure to an AMF. Reactions that were not exposed to an AMF expressed minimal mNG, and their fluorescence intensity was comparable to the inactive DNA template only. Reactions exposed to an AMF expressed mNG and their fluorescence was consistent with reactions containing the full mNG template (dsDNA T7 promoter region present). Representative of *n* = 3 biological replicates. Data are presented as mean values with error bars representing the s.d. **P < 0.02 (*P* = 0.016); NS, not significant (*P* = 0.30) from one-tailed *t*-tests. Source data

To control CFPS with magnetism, we synthesized an inactive DNA template encoding the fluorescent protein mNeonGreen (mNG) with a single-stranded 3ʹ overhang of the bottom T7 promoter sequence (on the template strand) and the top T7 promoter sequence (on the non-template strand) missing, taking advantage of T7 RNA polymerase only transcribing from a dsT7 promoter sequence and possessing the ability to traverse discontinuities in the non-template strand^41,42^. The T7 promoter released from magnetic hyperthermia was designed to hybridize to the inactive DNA template and restore the dsT7 promoter sequence, in turn allowing T7 RNA polymerase to bind, activating CFPS. To achieve this, we synthesized two mNG DNA templates by polymerase chain reaction (PCR), one with and one without the dsDNA T7 promoter region, and then used lambda exonuclease to generate the respective single-stranded versions to anneal together (Supplementary Fig. 6a,b). Lambda exonuclease preferentially degrades 5ʹ-phosphorylated strands of dsDNA. Therefore, asymmetrically 5ʹ-phosphorylated dsDNA can be degraded to leave a specific single-stranded DNA strand. As such a 5ʹ-phosphorylated primer was used for the mNG non-template strand with the promoter region and the mNG template strand without the promoter region, this enabled the digestion of the individual phosphorylated DNA strands by lambda exonuclease. The intact single-stranded mNG template with the promoter region and the intact single-stranded mNG non-template without the promoter region were annealed to produce the inactive mNG DNA template (Supplementary Fig. 6a,b). To calculate the loss of activity of the inactive template, we used a commercial CFPS system (PURExpress) and measured the fluorescence intensity of expressed mNG protein after incubation for 3 h. The inactive template had 6% activity compared with the full template containing the intact dsDNA T7 promoter region (Fig. 3b), whereas using no DNA template gave 2% activity, compared with the full template. We explored further the influence of the IONPs@SiO_2_ alone on mNG expression and saw minimal difference in the activity of PURExpress and the fluorescence intensity of mNG expression with and without the nanoparticles present (Supplementary Fig. 3b).

The inactive DNA template was combined with our magnetically activated SNAs (containing the T7 promoter sequence) to control CFPS with an AMF (Fig. 3a). The localized thermal energy dissipated from the magnetic nanoparticles under an applied AMF was hypothesized to denature the conjugated dsDNA and release the hybridized T7 promoter (top strand). The newly released top strand would then be free to hybridize to the single-stranded T7 promoter of the inactive template, restoring the dsDNA T7 promoter and activating CFPS of the encoded fluorescent mNG protein. We applied an AMF through an 18-turn solenoid coil (50 mm diameter) with parameters chosen to ensure operation within clinically tolerable frequencies (100 kHz)^14,25^. As such the AMF parameters selected were a field amplitude of 30 mT and a frequency of 103.4 kHz, ensuring further a magnetic field–frequency product (*H* × *f* = 2.47 × 10^9^ A m^−1^ s^−1^) below the safety limit proposed by both Hergt et al.^43^ (*H* × *f* = 5 × 10^9^ A m^−1^ s^−1^) and Herrero de la Parte et al.^44^ *(H* × *f* = 9.59 × 10^9^ A m^−1^ s^−1^). The timeframe was adjusted to ensure that the solution did not experience heating that was incompatible with the commercial CFPS system (PURExpress) and was set at 25 min AMF exposure, during which the temperature was measured by suspending a fibre-optic probe into the solution (Supplementary Fig. 7).

The magnetically activated SNAs, inactive mNG template and CFPS system were exposed to an AMF, with a control experiment without an applied AMF. The resulting mNG expression was inferred by the measured fluorescence intensity after incubation for 3 h and compared with the inactive template alone (negative control) and the full mNG DNA template containing the dsDNA T7 promoter region (positive control), representing a theoretical 100% restoration of activity (Fig. 3b). In the control without an applied AMF, we observed only a minor increase in fluorescence compared with the inactive template (significant, *P* = 0.016), which is probably due the small amount of electrostatically bound dsDNA T7 promoter left on the SNAs (Supplementary Fig. 4). In contrast, exposing the magnetically activated SNAs to an AMF in the presence of the inactive mNG template and the CFPS system yielded full recovery of expression compared with the full template (non-significant, *P* = 0.30), showing the tight regulation of biosynthesis in situ and the selective ‘on’ state only in the presence of the magnetic field. We carried out control CFPS reactions with and without IONPs@SiO_2_ nanoparticles, in the presence of the short dsDNA T7 promoter sequence (that was not connected to the nanoparticles), and with the inactive template. There was only a minor increase in mNG expression upon exposure to an AMF for 25 min at a magnetic field strength and frequency of 30 mT and 103.4 kHz, as compared with the identical CFPS but without exposure to an AMF. This demonstrates that the denaturing of the dsDNA T7 promoter sequence at the surface of the magnetically activated SNAs is a direct result of inductive heating from magnetic hyperthermia and not simply the heat generated from the solenoid coil (Supplementary Fig. 8a,b).

## Magnetic activation of gene-expressing synthetic cells

**Fig. 4:**
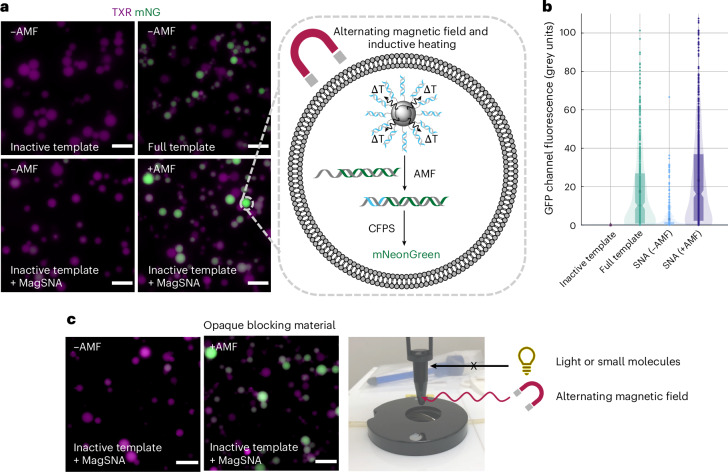
Controlling gene expression in synthetic cells with an alternating magnetic field. **a**, Epifluorescence microscopy images of magnetically activated SNAs and in situ mNG expression in synthetic cells with and without exposure to an AMF. Synthetic cells (visualized through the encapsulated TXR) that were not exposed to an AMF expressed minimal mNG, and their GFP channel fluorescence intensity was comparable to synthetic cells that contained PURExpress and the inactive DNA template only. Synthetic cells exposed to an AMF expressed mNG, as demonstrated by the increase in the green channel fluorescence, and were consistent with synthetic cells containing the full mNG template (dsDNA T7 promoter region present). Images are representative of *n* = 3 biological replicates. Scale bar, 20 μm. *T*, temperature. **b**, Quantification of mNG expression in the individual GUVs using a circle-detection-based image analysis script. Mean fluorescence intensity (inactive template), 0.00 grey units; mean fluorescence intensity (SNA, no AMF), 3.03 grey units; mean fluorescence intensity (full template), 17.47 grey units; and mean fluorescence intensity (SNA, AMF), 23.22 grey units. Datasets are representative of *n* = 3 biological replicates. The boxplot, notch and asterisk represent the interquartile range, median and mean fluorescence intensity, respectively. **c**, Epifluorescence microscopy images of magnetically activated SNAs and in situ mNG expression in synthetic cells within an opaque blocking material (black tube, see image). Synthetic cells that were not exposed to an AMF expressed minimal mNG, whereas synthetic cells exposed to an AMF expressed mNG, unperturbed by the blocking layer. Images are representative of *n* = 2 biological replicates. Scale bar, 20 μm. Source data

To demonstrate magnetic activation of CFPS-containing synthetic cells, we encapsulated the magnetically activated SNAs, inactive mNG template and CFPS system (PURExpress) inside giant unilamellar vesicles (GUVs). GUVs containing PURExpress were prepared as previously described, using egg phosphatidyl choline (egg-PC) and the inverted emulsion method^3^. It was important to choose a lipid, such as egg-PC, whose transition temperature was not within the range used for denaturing the DNA^45^. Briefly, an emulsion of the internal synthetic cell solution was prepared with a lipid-containing oil to form droplets stabilized by a lipid monolayer. The emulsion was then centrifuged through a phase-transfer column, consisting of the outer buffer solution below the same lipid-containing oil, forming another (second) lipid monolayer at the interface. The lipid-stabilized droplets were centrifuged across the second lipid monolayer phase-transfer column, forming a bilayer at their surface^3^. We encapsulated the PURExpress solution, magnetically activated SNAs, the inactive DNA template encoding mNG, and Texas-Red-Dextran (TXR) to visualize the resulting GUVs. After preparation, the GUVs underwent activation through exposure to an AMF (with a control not exposed to an AMF), followed by incubation for 2.5 h to enable CFPS, and finally imaging by fluorescence microscopy. A fibre-optic probe was used to measure the temperature of the solution, demonstrating that it did not exceed 33 °C during exposure to an AMF and that the bulk heating experienced was comparable both with and without the magnetically activated SNAs present (Supplementary Fig. 9). Release studies were carried out by replicating the CFPS conditions inside the synthetic cells and incubating the magnetically activated SNAs and a separate T7 promoter bottom strand in the CFPS buffer at multiple temperatures, and compared to AMF exposure. PicoGreen quantification of the released T7 top strand and subsequent annealing to the extra T7 bottom strand in solution demonstrated that the surface of the nanoparticles heated to around 45 °C upon AMF exposure (Supplementary Fig. 10). The synthetic cells encapsulating the magnetically activated SNAs and the inactive mNG template, but without exposure to an AMF, showed minimal expression (mean fluorescence intensity, 3.03 grey units), compared with the inactive template (mean fluorescence intensity, 0.00 grey units), demonstrating an excellent ‘off’ state (Fig. 4a,b). The in situ mNG expression inside the synthetic cells was fully recovered after exposure to an AMF, compared with the full template (mean fluorescence intensity, 23.22 versus 17.47 grey units). The enhanced mNG expression was observed only in the presence of the AMF, realizing the remote control of synthetic cells with an AMF.

The ability for magnetic fields to deeply penetrate bodily tissue is advantageous over previously published remote stimuli, predominantly light^3,46^. Moreover, magnetic hyperthermia is approved by the Food and Drug Administration and the European Medicines Agency for the treatment of glioblastomas, and the available clinical formulation NanoTherm (aminosilane-coated ferrofluid) is comparable to our central scaffold^25^. To demonstrate superiority over the state-of-the-art and the applicability of magnetic fields as a deeply penetrating external stimulus, we activated synthetic cells within an opaque blocking material (black tube) (Fig. 4c). These types of materials, being rigid polymers, have been used as mimics of hard tissues^47–49^ and act further as a blocking layer that is impenetrable to the current state-of-the-art remote stimuli: small molecules and light^3^. As was previously observed, there was minimal leakage of mNG expression in the absence of an AMF (mean fluorescence intensity, 5.06 grey units) (Fig. 4c and Supplementary Fig. 11). The ‘on’ state and the ability to control the magnetically activated SNAs and in situ mNG expression with exposure to an AMF was not perturbed by the blocking layer (mean fluorescence intensity, 24.55 grey units). The conservation of a high mNG fluorescence after exposure to an AMF in an opaque blocking material, which is otherwise impenetrable to current activation methods, lays the foundations for synthetic cells as controllable drug delivery devices.

## Magnetic control and cargo release from synthetic cells

To realize the utility of the system, we synthesized an inactive DNA template encoding the pore-forming protein α-haemolysin (α-HL) and exploited our magnetically activated SNAs and CFPS to selectively release a model cargo from synthetic cells. Initially, we synthesized the inactive DNA template encoding α-HL in an identical manner to that described for the inactive mNG template. Two α-HL DNA templates were synthesized by PCR: one containing the T7 promoter and one without. For the PCR with the promoter, a 5ʹ-phosphorylated primer was used for the α-HL non-template strand. For the PCR without the promoter, a 5ʹ-phosphorylated primer was used for the α-HL template strand. Following the digestion of the phosphorylated strands by lambda exonuclease, the remaining two strands were annealed to form the inactive α-HL DNA template (Supplementary Fig. 12).

**Fig. 5:**
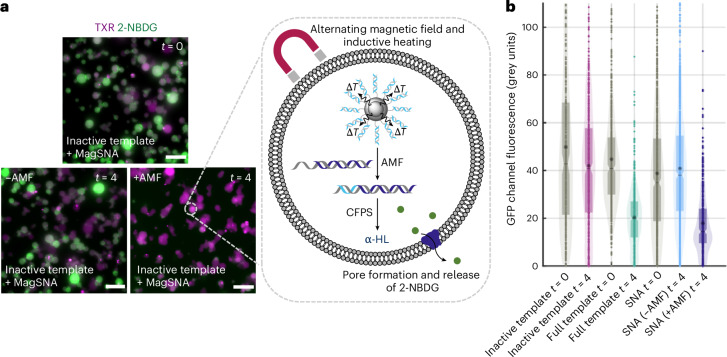
Controlling α-HL expression and cargo release from synthetic cells with an alternating magnetic field. **a**, Epifluorescence microscopy images of magnetically activated SNAs and in situ α-HL expression in synthetic cells with and without exposure to an AMF. Synthetic cells (visualized through the encapsulated TXR) that were not exposed to an AMF retained 2-NBDG fluorescence, indicative of minimal α-HL expression, preventing the formation of pores in the lipid bilayer and the leakage of 2-NBDG. Synthetic cells exposed to an AMF showed a significant loss of 2-NBDG and its fluorescence, representative of the in situ α-HL expression activated in the AMF. Images are representative of *n* = 4 biological replicates. Scale bar, 20 μm. *T*, temperature. **b**, Quantification of 2-NBDG fluorescence in the individual GUVs using a circle-detection-based image analysis script at *t* = 0 h and *t* = 4 h. Mean fluorescence intensity (full template), 44.72 grey units at *t* = 0 h and 20.33 grey units at *t* = 4 h; mean fluorescence intensity (inactive template), 49.80 grey units at *t* = 0 h and 41.97 grey units at *t* = 4 h; mean fluorescence intensity (SNA, no AMF), 38.81 grey units at *t* = 0 h and 40.90 grey units at *t* = 4 h; and mean fluorescence intensity (SNA, AMF), 17.92 grey units at *t* = 4 h. Datasets are representative of *n* = 4 biological replicates. The boxplot, notch and asterisk represent the interquartile range, median and mean fluorescence intensity, respectively. Source data

To demonstrate the magnetic activation of α-HL expression in synthetic cells, and subsequent release of a model cargo, we prepared GUVs using egg-PC and the inverted microemulsion method and encapsulated PURExpress solution, the previously developed magnetically activated SNAs, inactive α-HL template and a fluorescent small molecule 2-(*N*-(7-nitrobenz-2-oxa-1,3-diazol-4-yl)amino)-2-deoxyglucose (2-NBDG). Control GUVs were also prepared without the magnetically activated SNAs but with the inactive α-HL template alone (negative control) and with the full α-HL DNA template containing the dsDNA T7 promoter region (positive control, representing maximum release of cargo). The GUVs were imaged by fluorescence microscopy at *t* = 0 h, representing the maximum fluorescence intensity of 2-NBDG inside the synthetic cells. The GUVs were then exposed to an AMF (with controls not exposed to an AMF) for 10 min at a magnetic field strength and frequency of 30 mT and 103.4 kHz, followed by incubation for 4 h to facilitate α-HL CFPS, membrane insertion and cargo release. The release (or lack thereof) of 2-NBDG from the synthetic cells after *t* = 4 h was tracked by fluorescence microscopy. In the positive control, containing full α-HL DNA template, α-HL expression and cargo release was observed as the loss of 2-NBDG fluorescence (mean fluorescence intensity, 44.72 grey units at *t* = 0 h changing to 20.33 grey units at *t* = 4 h) (Fig. 5b and Supplementary Fig. 13). Whereas, for the negative control, using the inactive α-HL template alone, the 2-NBDG fluorescence was conserved in the synthetic cells (mean fluorescence intensity, 49.80 grey units at *t* = 0 h changing to 41.97 grey units at *t* = 4 h). For the synthetic cells encapsulating the magnetically activated SNAs and the inactive mNG template, but without exposure to an AMF, the 2-NBDG fluorescence was retained (mean fluorescence intensity, 38.81 grey units at *t* = 0 h changing to 40.90 grey units at *t* = 4 h), demonstrating a robust ‘off’ state without leakage of α-HL expression (Fig. 5a,b). However, following exposure to the AMF, the α-HL expression and release of 2-NBDG fluorescence was similar to the positive control (mean fluorescence intensity, 38.81 grey units at *t* = 0 h changing to 17.92 grey units at *t* = 4 h). This magnetically activated release of cargo from synthetic cells represents a stepping stone towards use for in vivo drug delivery.

## Conclusions

We have described a robust strategy for controlling CFPS inside synthetic cells using magnetic hyperthermia, a clinically approved and deeply tissue-penetrating stimulus, by generating magnetically activated SNAs. We further demonstrated an initial cargo release system by synthesizing the pore-forming protein α-HL on demand and only in the presence of a magnetic field. Beyond this, further nanoparticle engineering will allow for the exploration of the inherent properties of the core IONPs as contrast agents for MRI, introducing the potential application of synthetic cells in theragnostics^50^, and in magnetophoresis, controlling the movement of synthetic cells in vivo^51^. Future work may also focus on further improving the release efficiency by enhancing the specific heat absorption rate of the nanoparticles, which might be achieved in part through modulating the size and morphology or incorporating dopants^17^. The bulk heating observed in the AMF can also be mediated by exploring a pulsed regime with intermittent AMF exposure applied sequentially, previously demonstrated in vivo^52^. We demonstrate that the activity of nucleic acids and/or CFPS can be controlled with an AMF. The promising ‘off’ state, showing negligible expression without the magnetic field, was only achieved with our SNA purification technique, which will prove essential to realizing synthetic cells as targeted therapeutic modalities. Importantly, we have shown the selective control of CFPS in synthetic cells using frequencies compatible with the only clinically available magnetic hyperthermia system by MagForce, optimized to minimize eddy currents within the body and reduce patient discomfort^25^. The in situ production of active biomolecules avoids side effects caused by drug leakage during storage and/or in circulation, which may arise from preloading vesicles^53^. The ability to control the activity of synthetic cells within an opaque blocking material renders the developed technology advantageous over small molecule or light activation, which are the current state-of-the-art. The next steps to apply this system will be to test the stability in a sophisticated tissue model^54^ and then the targeting activity in vivo^5–7^. CFPS-containing synthetic cells have already been applied in mouse models, and are of interest as potential cancer therapeutics^7^. The modular nature of our system will allow for more pharmaceutically relevant proteins to be accessed by simply changing the inactive DNA template. Furthermore, the lipid bilayer can be functionalized with targeting ligands^55^, to increase synthetic cell concentration at the target site, and stealth polymers^56–58^, to improve stability and circulation. This selective regulation of biosynthesis in situ demonstrates the remote control of synthetic cells using deeply tissue-penetrating magnetic fields and lays the foundations for the application of synthetic cells as smart delivery devices.

## Methods

### Materials

All solvents and reagents were purchased from Sigma/Merck unless stated otherwise. Iron acetylacetonate was purchased from Fluorochem. Dibenzocyclooctyne-*N*-hydroxysuccinimidyl (DBCO-NHS) ester was purchased from Cambridge Bioscience. DreamTaq DNA polymerase master mix, TXR (10 kDa) and 25-μl gene frames were purchased from Thermo Fisher Scientific. PURExpress, Lambda Exonuclease, Purple Loading Dye (6×) and RNA Gel Loading Dye (2×) was purchased from New England Biolabs. Egg-PC was purchased from Avanti Polar Lipids. T7 promoter sequences (azide and non-modified) were synthesized by Integrated DNA Technologies.

### Characterization

DLS was performed with a Zetasizer-Nano-ZS (Malvern Instruments) at 25 °C with nanoparticle solution at a concentration of 50 mg l^−1^ to obtain the hydrodynamic diameter, PDI and *ζ*-potential.

XRD patterns were obtained using a Xcalibur PX Ultra diffractometer equipped with a Co Kα radiation source (*λ* = 1.79 Å) operated at 40 mA. The crystallite diameter was evaluated by considering the X-ray wavelength, *λ* = 0.154 nm, with the crystallite-shape factor *K* set to 0.9, and the line broadening at the full half-width maximum of the most intense peak (in radians) and the Bragg angle (*θ* = 0.31 rad)^59^.

$${{D}}_{{\rm{XRD}}}=\frac{{K}{\lambda }}{{\beta }\cos \left({\theta}\right)}$$

TEM images were captured using JEOL CX100 II TEM at 100 kV. Nanoparticle dispersions (0.1 mg ml^−1^ in ethanol) were drop-cast onto carbon-coated copper grids and air-dried at room temperature. Particle size analysis was performed on >100 particles using ImageJ software.

The magnetic properties were characterized using a MPMS-5S superconducting quantum interference device (SQUID) magnetometer (Quantum Design).

Alternating magnetic fields were generated using NAN201003 Magnetherm (Nanotherics) with an 18-turn coil of 50 mm internal diameter and at a magnetic field strength of 30 mT and a frequency of 103.4 kHz.

### Synthesis of oleylamine-capped iron oxide nanoparticles

Iron acetylacetonate (1.0595 g, 3 mmol) was dissolved in 15 ml of benzyl ether and 15 ml of oleylamine. The reaction was evacuated and filled with argon five times (the vacuum was held for 5 min per cycle) and the reaction vessel was purged further with argon for 5 min to remove any remaining moisture. The solution was dehydrated at 110 °C from room temperature (3 °C min^−1^, 37 min) for 1 h under an argon atmosphere, before being heated to 293 °C (20 °C min^−1^, 9.5 min) and aged at this temperature for 1 h. The solution was magnetically stirred (500 r.p.m.) throughout the synthesis. Ethanol (40 ml) was subsequently added to the reaction mixture and the black material was precipitated and separated via centrifugation (4,297*g*, 10 min). The precipitate was dissolved in cyclohexane. Undispersed residue was removed by centrifugation (4,297*g*, 10 min). The product was precipitated with ethanol and centrifuged (4,297*g*, 10 min) to remove the solvent, and redispersed into cyclohexane. The resulting oleylamine-capped iron oxide nanoparticles were stored at 4 °C in cyclohexane^29,30^.

### Silica encapsulation and amine modification of iron oxide nanoparticles

IGEPAL-*co*-520 (0.5 ml, 0.5 g, 1 mmol) was dispersed in cyclohexane (11 ml, 101 mmol) and injected with oleylamine-capped iron oxide nanoparticles dispersed in cyclohexane (1 ml, 15 mg ml^−1^). The dispersion was sonicated for 10 min at ambient conditions. Ammonium hydroxide 1 M (0.13 ml, 0.13 mmol) was added to a magnetically stirred solution (600 r.p.m.). The controlled addition of tetraethylorthosilicate (TEOS) (317 μl, 1.42 mmol) was performed using the fractionated drop-method, with a known volume of TEOS (39.6 μl) being added every 3 h. A total of three additions were carried out per day and reacted overnight, whereby the fourth addition was carried out 24 h after the first addition and the seventh addition was carried out 48 h after the first addition. Three hours after the eighth addition, (3-aminopropyl)triethoxysilane (APTES) (39.4 μl, 0.17 mmol) was added and the reaction was left overnight. The reaction was then diluted with ethyl acetate. Centrifugation (4,297*g*, 10 min) was applied to pellet the nanoparticles and the solvent was removed. The nanoparticles were then dispersed in ethanol (1 part) by sonication and diluted with ethyl acetate (3 parts) before pelleting by centrifugation (4,297*g*, 10 min) and removing the solvent. This step was repeated twice more to remove the IGEPAL-*co*-520. The resulting silica-encapsulated iron oxide nanoparticles were then dispersed in ethanol (1 ml) to give a concentration of 15 mg ml^−1^ and stored at 4 °C (ref. ^34^).

### Modification of amine-modified silica-encapsulated iron oxide nanoparticles

Amine-modified silica-encapsulated iron oxide nanoparticles (300 μl, 5 mg ml^−1^) were redispersed in 3-morpholinopropane-1-sulfonic acid (MOPS) buffer (0.1 M, pH 7.4). To this, DBCO-NHS ester (150 μl, 100 mM in dimethylformamide (DMF)) was added. The resulting dispersion was briefly sonicated and incubated overnight at room temperature in a ThermoMixer C with shaking at 1,000 r.p.m. The DBCO-modified silica-encapsulated iron oxide nanoparticles were purified by centrifugation (17,000*g*, 10 min), washing three times in ethanol, and stored in ethanol (5 mg ml^−1^) at 4 °C.

### Synthesis of spherical nucleic acids

The 5ʹ-azide-modified reverse compliment of the T7 promoter was annealed to the T7 promoter sequence in equimolar concentrations prior to heating at 95 °C for 5 min (and cooling to room temperature over the course of 1 h) and analysis by PAGE. A native 16% (v/v) PAGE was set up on a 5-ml scale containing 40% (w/v) acrylamide/bisacrylamide solution (2 ml), 10× TBE buffer (0.5 ml), water (2.5 ml), ammonium persulfate (40 μl, 10% w/v) and TEMED (2.5 μl). The reaction was briefly vortexed and left to set for 30 min. The samples were prepared by mixing the DNA (180 ng, 10 μl) in Native Loading Dye (2×, 10 μl). Gels were run with 1× TBE buffer at 250 V for 60 min and then stained in 3× Gel Red for 10 min. Gels were imaged using Azure 200 Gel Imager (Azure Biosystems).

DBCO-modified silica-encapsulated iron oxide nanoparticles (5 mg ml^−1^, 50 μl) were dispersed in water (395.9 μl). Sodium chloride was added (300 mM, 37.50 μl of 4 M stock solution). The resulting dispersion was sonicated briefly. To this, azide-modified dsT7 promoter (3 μM, 16.6 μl of 90.55 μM stock solution) was added and the reaction was vortexed for several seconds. The reaction was then placed at −7 °C (Optima LTC4R refrigerated bath, Grant Instruments) for 4 h and then in a −20 °C freezer overnight before thawing to room temperature for 1 h at 4 °C (ref. ^39^). The resulting T7-promoter-modified silica-encapsulated iron oxide nanoparticles (magnetically activated SNAs) were purified by centrifugation at 17,000*g* for 13 min. The pellet was redissolved in a minimal volume of water (<30 μl). The magnetically activated SNAs were purified by agarose gel electrophoresis, whereby 6× purple loading dye (5 μl) was added and the reaction briefly vortexed. A 1.5% (w/v) TBE agarose gel was prepared by dissolving LE agarose (0.75 g) in 3× Gel Red (17 ml), 10× TBE buffer (5 ml) and water (28 ml) in a microwave (mid-high at intervals of 20 s). The gel was left to set for 30 min before the aforementioned magnetically activated SNAs were loaded into the wells and the gel was run for 20 min at 110 V to pull off any non-covalently bound azide-modified dsT7 promoter. The well with the magnetically activated SNAs was excised and briefly sonicated to release the purified SNAs. The purified magnetically activated SNAs were pelleted by centrifugation at 17,000*g* for 13 min and redissolved in water.

### Synthesis of the inactive mNG template

DreamTaq MM (2×) (60 μl) was mixed with phosphorylated T7 forward primer or −T7 forward primer (2.4 μl, 25 μM), CT-rev or phosphorylated CT-rev, respectively (2.4 μl, 25 μM), mNG PURExpress control template plasmid^6^ (1.2 μl, 2 ng μl^−1^) and water (54 μl). Reactions were cycled according to the following programme using a ProFlex PCR System (ThermoFisher): 95 °C for 60 s, 35 cycles (95 °C for 30 s, 49 °C for 30 s and 72 °C for 65 s), 72 °C for 10 min, 4 °C hold. PCR products were validated by 1.5% (w/v) TBE agarose gel electrophoresis and purified using a Monarch PCR & DNA Cleanup Kit (New England Biolabs).

Double-stranded mNG templates with and/or without the dsT7 promoter region (1,000 ng, 10.66 μl) were diluted with water (11.34 μl). Lambda exonuclease (0.5 μl) and lambda exonuclease reaction buffer (10×, 2.5 μl) was added to a final volume of 25 μl. The reaction was allowed to proceed at 37 °C for 30 min (template without the dsT7 promoter) and 35 min (template with the T7 promoter) before the lambda exonuclease was denatured at 70 °C for 10 min. The crude reaction mixtures were combined and annealed at 95 °C for 5 min (before slow cooling to room temperature). The resulting inactive mNG template was purified using a Monarch PCR & DNA Cleanup Kit and the purity was validated by 1.5% (w/v) TBE agarose gel electrophoresis.

The inactive α-HL template was synthesized using the same protocol but with a PURExpress control template plasmid encoding the α-HL protein.

### Bulk cell-free protein synthesis of mNG protein

Cell-free protein synthesis reactions (3 μl) were prepared with PURExpress Solution A (1.2 μl), PURExpress Solution B (0.9 μl) and supplemented with murine RNase inhibitor (0.075 U μl^−1^). For the negative control, inactive mNG template (5 ng μl^−1^, 0.30 μl) and water (0.52 μl) were added; for the positive control the full mNG template (with the dsT7 promoter present) (5 ng μl^−1^, 0.30 μl) and water (0.52 μl) were added; and for the magnetically activated SNA samples, inactive mNG template (5 ng μl^−1^, 0.30 μl), magnetically activated SNAs (200 nM (as measured by calibration curve—Supplementary Fig. 5), 0.30 μl) and water (0.22 μl) were added. If necessary, the reactions were exposed to an alternating magnetic field (103.4 kHz, 30 mT) for 25 min and left for a further 25 min at room temperature. All reactions were then incubated in a thermal cycler at 25 °C for 3 h and worked up with 17 μl of water. Reactions were transferred to Perkin Elmer 384-well black flat-bottom OptiPlates. Fluorescence was measured using a Tecan Spark fluorescence plate reader (Tecan Group) (bandwidth, 5 nm; *z* position, 17,764 μm; excitation mNG, 495 nm; emission mNG, 517 nm).

Control CFPS reactions were carried out with the same protocol but the magnetically activated SNAs were replaced with dsDNA T7 promoter (200 nM, 0.30 μl). Similarly, in the control with DBCO-modified IONPs@SiO_2_ present (free in solution), SNAs were replaced with dsDNA T7 promoter (200 nM, 0.30 μl) and additional DBCO-modified IONPs@SiO_2_ were added (1.5 mg ml^−1^, 0.30 μl).

### Assembly of mNG and magnetic activation of synthetic cells

Egg-PC dissolved in chloroform (25 mg ml^−1^, 150 μl) was transferred to a 2-ml glass vial (per two conditions). The vials were tilted at 45° and rotated slowly while held under N_2_ flow to distribute the lipids evenly up the walls. Vials containing lipid films were held under a vacuum for 3 h to remove residual chloroform. Mineral oil (filtered through 0.22-μm PES membrane) was added to egg-PC films (0.647 g) to give a final concentration of 5 mg ml^−1^ egg-PC. Vials were vortexed for 1 min and then incubated in an oven at 80 °C for 10 min with the lids removed. Lids were reapplied to vials and sealed tightly using Teflon tape and parafilm, then vortexed aggressively for 1 min and sonicated in a sonication bath for 1 h at 50 °C. Lipid-containing oil was stored at room temperature overnight and vortexed, then sonicated for 10 min at room temperature immediately before use. A total of 250 μl of 5 mg ml^−1^ egg-PC in mineral oil was transferred to 1.5-ml centrifuge tubes and placed on ice. A total of 10 μl inner solution (PURExpress containing 5 ng μl^−1^ inactive mNG template or full mNG template, 400 nM magnetically activated SNAs (as measured by calibration curve—Supplementary Fig. 5), 1.0 U μl^−1^ murine RNase inhibitor, 25 μM TR-dextran (10 kDa) and 200 mM sucrose) was added into the chilled lipid-containing oil, ensuring the tip was constantly moved through the lipid-containing oil to disperse the inner solution. Tubes were passed across a centrifuge rack once using light pressure to form cloudy water-in-oil (W/O) emulsions. Meanwhile, 100 μl of lipid-containing oil was layered on top of 250 μl of chilled outer solution (50 mM HEPES, 400 mM potassium glutamate and 200 mM glucose, pH 7.6) and placed at room temperature. W/O emulsions were then added on top of this oil phase, and this tube was left at room temperature for 1 min. Centrifuge tubes containing the W/O emulsion above the outer solution were centrifuged at 16,000*g*, 4 °C, for 30 min. After centrifugation, the oil phase and ~200 μl of the outer solution were removed from the tube and discarded. Using a fresh tip, ~10 μl of the remaining outer solution was ejected against the GUV pellet to displace it from the tube, and the intact GUV pellet was transferred to a new tube containing 250 μl outer solution. The pellet was subsequently resuspended by gently pipetting up and down. Vesicles were centrifuged at 10,000*g*, 4 °C for 10 min, then the outer solution was removed and the pellets were gently resuspended in 25 μl of fresh outer solution^6^.

We assembled the α-HL-containing synthetic cells using the same protocol, but instead the inner solution consisted of PURExpress containing 5 ng μl^−1^ inactive α-HL template or full α-HL template, 400 nM magnetically activated SNAs (as measured by calibration curve—Supplementary Fig. 5), 1.0 U μl^−1^ murine RNase inhibitor, 25 μM TR-dextran (10 kDa), 250 μM 2-NBDG and 200 mM sucrose.

### mNG expression in synthetic cells

To assess mNG expression from inside the synthetic cells, the GUV pellets were resuspended in 25 μl outer solution and placed in colourless or black (optical blocking material) microcentrifuge tubes. If necessary, the synthetic cells were exposed to an alternating magnetic field (103.4 kHz, 30 mT) for 10 min and left for a further 10 min at room temperature. All reactions were then incubated in a thermal cycler at 25 °C for 2.5 h and worked up with 25 μl of outer solution (50 mM HEPES, 400 mM potassium glutamate and 200 mM glucose, pH 7.6). Fluorescence microscopy was performed with an EVOS FL fluorescence microscope using a ×100 oil immersion objective lens. GUVs were imaged using bright-field, TXR and GFP filters.

We assessed the synthesis of the α-HL pore-forming protein, insertion in the lipid membrane and release of the 2-NBDG small molecule by resuspending the GUV pellets in 50 μl of outer solution and splitting each condition (inactive template, full template and inactive template/magnetically activated SNAs) into two centrifuge tubes. One centrifuge tube for each GUV sample (25 μl) was imaged for time point *t* = 0 h using an EVOS FL fluorescence microscope (×100 oil immersion objective lens). GUVs were imaged using bright-field, TXR and GFP filters. The other centrifuge tube for the sample containing the inactive template/magnetically activated SNAs (25 μl) was exposed to an alternating magnetic field (103.4 kHz, 30 mT) for 10 min and left for a further 10 min at room temperature. All reactions (including the three *t* = 0 h samples and the sample exposed to an AMF) were then incubated in a thermal cycler at 25 °C for 4 h. The reactions were imaged for time point *t* = 4 h with an EVOS FL fluorescence microscope (×100 oil immersion objective lens).

### Image processing

TXR and GFP channel brightness was normalized across all images within a single experiment in ImageJ (an open-source platform: https://imagej.net/licensing/open-source), and then the separate channels were saved as individual PNG files. All images corresponding to a single sample were stored within the same directory. ‘Background’ images were created by manually selecting vesicle-free regions of microscopy images (one from each sample within the experiment) and measuring the mean pixel intensity. PNG files were input into a vesicle analysis script (the original script can be found at https://zenodo.org/record/7729425)^3^.

## Online content

Any methods, additional references, Nature Portfolio reporting summaries, source data, extended data, supplementary information, acknowledgements, peer review information; details of author contributions and competing interests; and statements of data and code availability are available at 10.1038/s41557-025-01909-6.

## Supplementary information

Supplementary Information Supplementary Figs. 1–13, supplementary calculations, and DNA and primer sequences.

## Source data

Source Data Fig. 1 Numerical data representing the UV-Vis spectrum for the DBCO-modified IONPs@SiO_2_.

Source Data Fig. 2 An uncropped image of the GelRed® stained 1.5 (w/v)% TBE agarose gel showing the agarose purification of the magnetically-activated SNAs.

Source Data Fig. 3 Numerical data representing the fluorescence intensity from n = 3 biological repeats of the experiments controlling cell-free protein synthesis with magnetic hyperthermia.

Source Data Fig. 4 Numerical data indicating the mean GFP channel intensity of individual GUVs that were identified using the vesicle analysis script.

Source Data Fig. 5 Numerical data indicating the mean GFP channel intensity of individual GUVs that were identified using the vesicle analysis script.

## Data Availability

All the data generated in this study are available within the Article, the Supplementary Information, figures and source data. Source data for the supplementary figures are available from Zenodo via https://zenodo.org/records/15692133 (ref. ^60^). Source data are provided with this paper.

## Code availability

The custom vesicle analysis script used in this study is available from Zenodo via https://zenodo.org/records/15692133 (ref. ^60^).
