# Supplementary material for: Magnetic activation of spherical nucleic acids enables the remote control of synthetic cells
Source: Nat Chem. 2025 Sep 2;17(10):1505–13. doi: 10.1038/s41557-025-01909-6 (PMC12491065; doi:10.1038/s41557-025-01909-6)
Supplement: Supplementary file 1 — Supplementary Figs. 1–13, supplementary calculations, and DNA and primer sequences. [file 41557_2025_1909_MOESM1_ESM.pdf]

nature chemistry

Supplementary information

https://doi.org/10.1038/s41557-025-01909-6

Magnetic activation of spherical nucleic acids enables the remote control of synthetic cells

In the format provided by the authors and unedited

Table of Contents

DNA Sequences **2**

Calculations **3**

Determination of nanoparticle concentration.. 3

Determination of DBCO loading 4

Determination of DNA loading 4

Supplementary Figures **5**

Fig. S1. Characterisation of oleylamine-capped IONPs. 5

Fig. S2. PAGE of DNA annealing. 6

Fig. S3. Effect of SNA purification by agarose gel and presence of nanoparticles in cell-free expression. 7

Fig. S4. Calibration curve to determine effectiveness of agarose purification. 8

Fig. S5. Calibration curve to determine SNA T7 promoter loading. 9

Fig. S6. Synthesis of the inactive mNG template. 10

Fig. S7. Fibre optic probe data for the global heating experienced by bulk cell-free protein synthesis reactions in the alternating magnetic field. 11

Fig. S8. Cell-free protein synthesis controls with an alternating magnetic field. 12

Fig. S9. Fibre optic probe data for the global heating experienced by the synthetic cells in the alternating magnetic field. 13

Fig. S10. Release studies of the magnetically-activated SNAs. 14

Fig. S11. Quantification of mNG expression in an opaque blocking material. 15

Fig. S12. Synthesis of the inactive α-HL template. 16

Fig. S13. Alpha-hemolysin expression and small molecule cargo release from synthetic cells. 17

References **23**

DNA Sequences

DNA name	DNA sequence	Modification
T7 Promoter Sequence (Top Strand)	GAAATTAATACGACTCACTATAG	
T7 Promoter Sequence (Bottom Strand)	CTATAGTGAGTCGTATTAATTTC	5′-azide C6 modifier
-T7 Forward Primer (mNG)	GTTTAACTTTAAGAAGGAGGTATACATATGGTGAG	
Phosphorylated CT-Rev	GATATAGTTCCTCCTTTCAG	5′-phosphorylated
Phosphorylated T7 Forward Primer	GAAATTAATACGACTCACTATAGGGTCTAG	5′-phosphorylated
CT-Rev	GATATAGTTCCTCCTTTCAG	
mNG Linear Template	gaaattaatacgactcactatagggtctagaaataattttgtttaactttaagaaggaggtatac<br>atATGGTGAGCAAAGGCGAAGAGGATAATATGGCAAGC<br>CTGCCTGCAACACATGAACTGCATATTTTTGGTAGCAT<br>TAACGGCGTGGATTTTGATATGGTTGGTCAAGGCACCG<br>GTAATCCGAATGATGGTTATGAAGAACTGAATCTGAA<br>AAGCACCAAAGGCGATCTGCAGTTTAGCCCGTGGATTC<br>TGGTTCCGCATATTGGTTATGGTTTTCATCAGTATCTGC<br>CGTATCCGGATGGTATGAGCCCGTTTCAGGCAGCAATG<br>GTTGATGGTAGCGGTTATCAGGTTCATCGTACCATGCA<br>GTTTGAAGATGGTGCAAGCCTGACCGTTAATTATCGTT<br>ATACCTATGAAGGCAGCCACATTAAAGGTGAAGCACA<br>GGTTAAAGGTACAGGTTTTCCGGCAGATGGTCCGGTTA<br>TGACCAATAGTCTGACCGCAGCAGATTGGTGTCGTAGC<br>AAAAAAACCTATCCGAACGATAAAACCATCATCAGCA<br>CCTTCAAATGGTCATATACCACCGGCAATGGTAAACGT<br>TATCGTAGCACCGCACGTACCACCTATACCTTTGCAAA<br>ACCGATGGCAGCAAACTATCTGAAAAATCAGCCGATG<br>TATGTGTTTCGCAAAACGGAACTGAAACATTCCAAAAC<br>CGAGCTGAACTTTAAAGAATGGCAGAAAGCATTTACC<br>GATGTGATGGGTATGGATGAGCTGTACAAATAATGAgg<br>atcccgggaattctcgagtaaggttaacctgcaggaggcctttaattaaggtggtgcggcc<br>gcgctagcggtcccgggggatcgatccggctgctaacaaagcccgaaaggaagctgagt<br>tggctgctgccaccgctgagcaataactagcataaccccttggggcctctaaacgggtctt<br>gaggggttttttgctgaaaggaggaactatatc	
α-HL Linear Template	TAATACGACTCACTATAGGGTCTAGAAATAATTTTGTT<br>TAACTTTAAGAAGGAGGTATACATATGGCAGATTCTGA<br>TATTAATATTAAAACCGGTACTACAGATATTGGAAGCA<br>ATACTACAGTAAAAACAGGTGATTTAGTCACTTATGAT<br>AAAGAAAATGGCATGCACAAAAAAGTATTTTATAGTTT<br>TATCGATGATAAAAATCACAATAAAAAACTGCTAGTTA<br>TTAGAACAAAAGGTACCATTGCTGGTCAATATAGAGTT<br>TATAGCGAAGAAGGTGCTAACAAAAGTGGTTTAGCCT<br>GGCCTTCAGCCTTTAAGGTACAGTTGCAACTACCTGAT	

	AATGAAGTAGCTCAAATATCTGATTACTATCCAAGAAA TTCGATTGATACAAAAAACTATATGAGTACTTTAACTT ATGGATTCAACGGTAATGTTACTGGTGATGATACAGGA AAAATTGGCGGCCTTATTGGTGCAAATGTTTCGATTGG TCATACACTGAACTATGTTCAACCTGATTTCAAAACAA TTTTAGAGAGCCCAACTGATAAAAAAGTAGGCTGGAA AGTGATATTTAACAATATGGTGAATCAAAATTGGGGAC CATACGATCGAGATTCTTGGAACCCGGTATATGGCAAT CAACTTTTCATGAAAACTAGAAATGGTTCTATGAAAGC AGCAGATAACTTCCTTGATCCTAACAAAGCAAGTTCTC TATTATCTTCAGGGTTTTCACCAGACTTCGCTACAGTTA TTACTATGGATAGAAAAGCATCCAAACAACAAACAAA TATAGATGTAATATACGAACGAGTTCGTGATGATTACC AATTGCATTGGACTTCAACAAATTGGAAAGGTACCAAT ACTAAAGATAAATGGACAGATCGTTCTTCAGAAAGAT ATAAAATCGATTGGGAAAAAGAAGAAATGACAAATTA ATGAGGATCCCGGGAATTCTCGAGTAAGGTTAACCTGC AGGAGGCCTTTAATTAAGGTGGTGCGGCCGCGCTAGC GGTCCCGGGGGATCGATCCGGCTGCTAACAAAGCCCG AAAGGAAGCTGAGTTGGCTGCTGCCACCGCTGAGCAA TAACTAGCATAACCCCTTGGGGCCTCTAAACGGGTCTT GAGGGGTTTTTTG	
-T7 Forward Primer (α-HL)	ATGGCAGATTCTGATATTAATATTAAAAC	

Calculations

Determination of nanoparticle concentration

The nanoparticle concentration (particles mL^{-1}) was calculated according previous literature[1] by approximating each individual IONP@SiO_2 as spherical in shape. The mass of the core-shell IONPs@SiO_2 ($m_{c\text{-}s}$) was estimated using the radii of both the core IONPs (r_c) and core-shell IONPs@SiO_2 ($r_{core\text{-}shell}$) from TEM analysis, and the known densities of silica ($\rho = 2.20$ g cm^{-3}) and magnetite ($\rho = 5.24$ g cm^{-3}) (Eq. S1–3). The concentration of the core-shell IONPs@SiO_2 ($N_{core\text{-}shell}$) was calculated from the mass concentration of nanoparticles in mg mL^{-1} (M_C) and the mass of the individual core-shell IONPs@SiO_2 ($m_{core\text{-}shell}$) (Eq. S4).

$$m_{core} = \frac{3}{4}\pi\, r_{core}^{3}\, \rho_{magnetite} \qquad \text{Eq. S1}$$

$$m_{shell} = \frac{3}{4}\pi\left(r_{core-shell}^{3} - r_{core}^{3}\right)\rho_{silica} \qquad \text{Eq. S2}$$

$$m_{core-shell} = m_{core} + m_{shell} \qquad \text{Eq. S3}$$

$$N_{core-shell} = \frac{M_C}{m_{core-shell}} \qquad \text{Eq. S4}$$

Determination of DBCO loading

The DBCO concentration (c) was calculated using the Beer Lambert Law (Eq. S5) from the absorbance (A) of the DBCO at 309 nm (taken on a NanoPhotometer C40 UV/Vis Spectrophotometer (Implen)), the molar extinction coefficient (ε) of DBCO at $\varepsilon = 12{,}000\ M^{-1}cm^{-1}$ (309 nm)[2], and a path length (l) of 0.01 cm.

$$A = \varepsilon cl \qquad \text{Eq. S5}$$

The DBCO concentration was then converted to moles and Avogadro's number (N_A) was used to convert to number of DBCO molecules in a known volume (N) (Eq. S6). The number of known DBCO molecules was divided by the number of nanoparticles present (calculated as described previously) to gain the number of DBCO molecules per nanoparticle.

$$N = N_A \times \text{mol} \qquad \text{Eq. S6}$$

Determination of DNA loading

DNA concentration on the SNAs was determined by denaturing the attached dsDNA at 95°C for 5 min in RNA Gel Loading Dye (2X) and comparing the concentration (and further moles) of DNA released to a calibration curve of known DNA concentrations (Fig S5). Following this, the concentration of DNA strands was calculated using Avogadro's number (N_A) (Eq. S6) and divided by the number of nanoparticles present.

Supplementary Figures

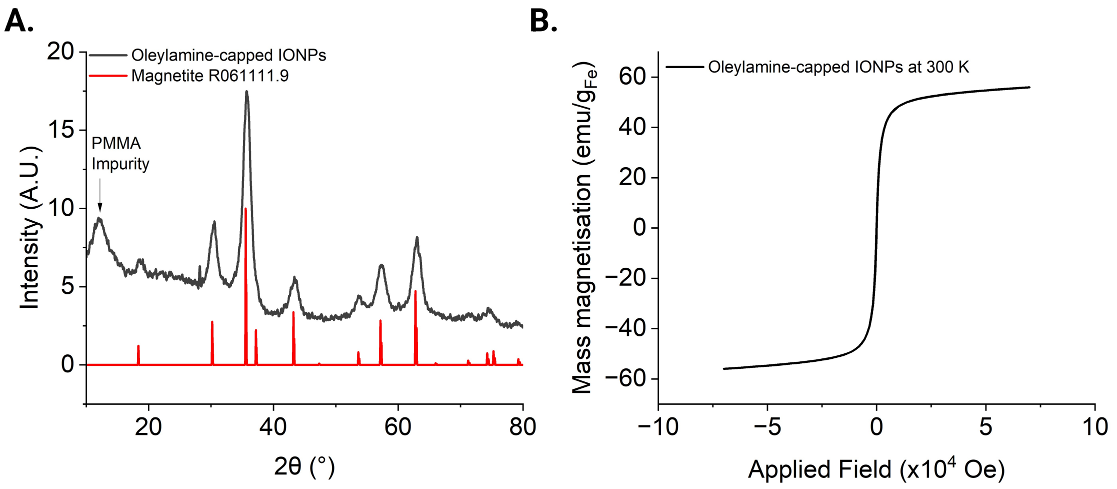

Fig. S1. Characterisation of oleylamine-capped IONPs. (A) XRD pattern of oleylamine-capped IONPs indexed against magnetite (RUFF ID:R061111). **(B)** Hysteresis curve of oleylamine-capped IONPs measured at 300 K.

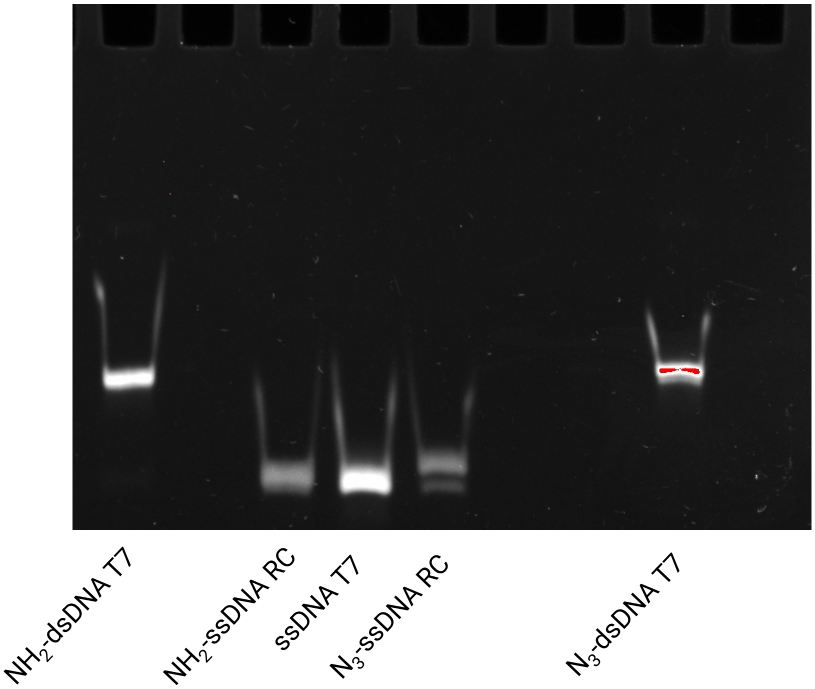

Fig. S2. PAGE of DNA annealing. Native 16% (v/v) poly(acrylamide) gel electrophoresis tracking the successful annealing of the T7 promoter bottom strand with (N_3-ssDNA RC) and without (NH_2-ssDNA RC) the N_3 click handle to the T7 promoter top strand (ssDNA T7), and the resulting dsDNA with (N_3-dsDNA T7) and without (NH_2-dsDNA T7) the N_3 click handle. The dsDNA is of higher molecular mass and so travels slower down the gel in the direction of the electric current (top to bottom of the gel).

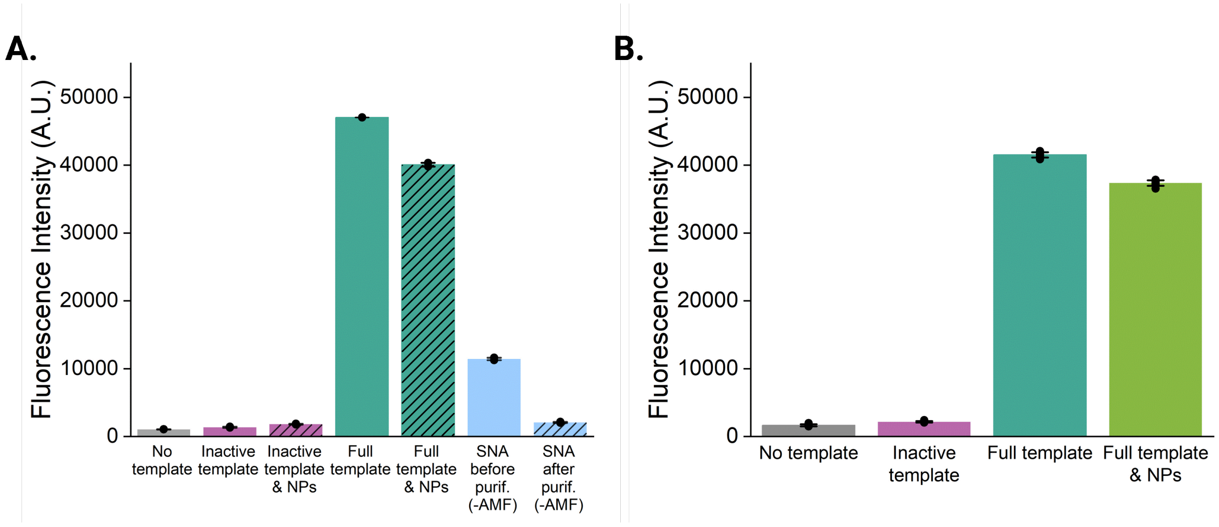

Fig. S3. Effect of SNA purification by agarose gel and presence of nanoparticles in cell-free expression. (A) In-vitro transcription of the Broccoli RNA aptamer that after expression binds to the small molecule DFHBI and fluoresces. RNA expression was recovered with the full Broccoli template compared to the inactive Broccoli template (without the ds T7 promoter) and the RNA expression and fluorescence intensity was not perturbed by the addition of NH_2-modified IONPs@SiO_2. The tight "off" state of the SNA (-AMF), comparable to the inactive template (negative control), was only achieved after agarose purification of the SNAs. Datasets are representative of n = 8 technical replicates. Data is presented as mean values +/– SD. **(B)** Cell-free protein synthesis of mNG in the presence of NH_2-modified IONPs@SiO_2, showing negligible inhibition of mNG expression and fluorescence intensity compared with the full mNG template (dsDNA T7 promoter region present) without the nanoparticles present. Datasets are representative of n = 8 technical replicates. Data is presented as mean values +/– SD.

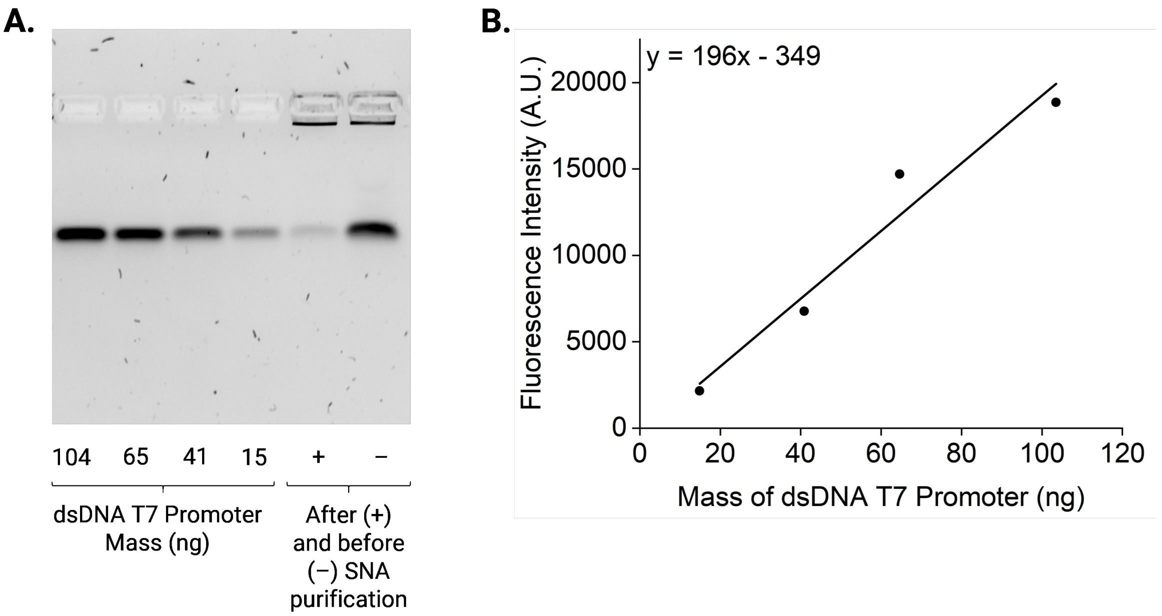

Fig. S4. Calibration curve to determine effectiveness of agarose purification. (A) 1.5% (w/v) agarose gel of known concentrations of the dsT7 promoter sequence and a known volume of SNA before and after agarose purification. **(B)** Calibration curve of the Gel Red fluorescence intensities (determined by ImageJ) against the known dsT7 promoter concentration, the linear regression (fitted in Origin) was used to extrapolate the concentration of dsT7 promoter electrostatically-bound to the SNA before and after agarose purification.

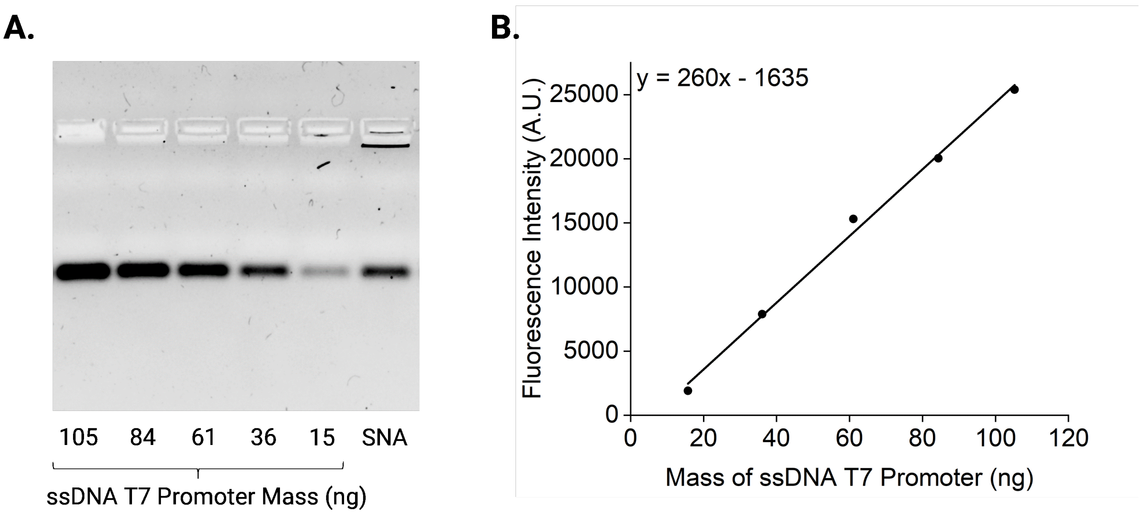

Fig. S5. Calibration curve to determine SNA T7 promoter loading. (A) 1.5% (w/v) agarose gel of known concentrations of the T7 promoter sequence and a known volume of SNA heated at 95°C in urea-containing dye to release the bound T7 promoter. **(B)** Calibration curve of the Gel Red fluorescence intensities (determined by ImageJ) against the known T7 promoter concentration, the linear regression (fitted in Origin) was used to extrapolate the concentration of bound T7 promoter on the SNA.

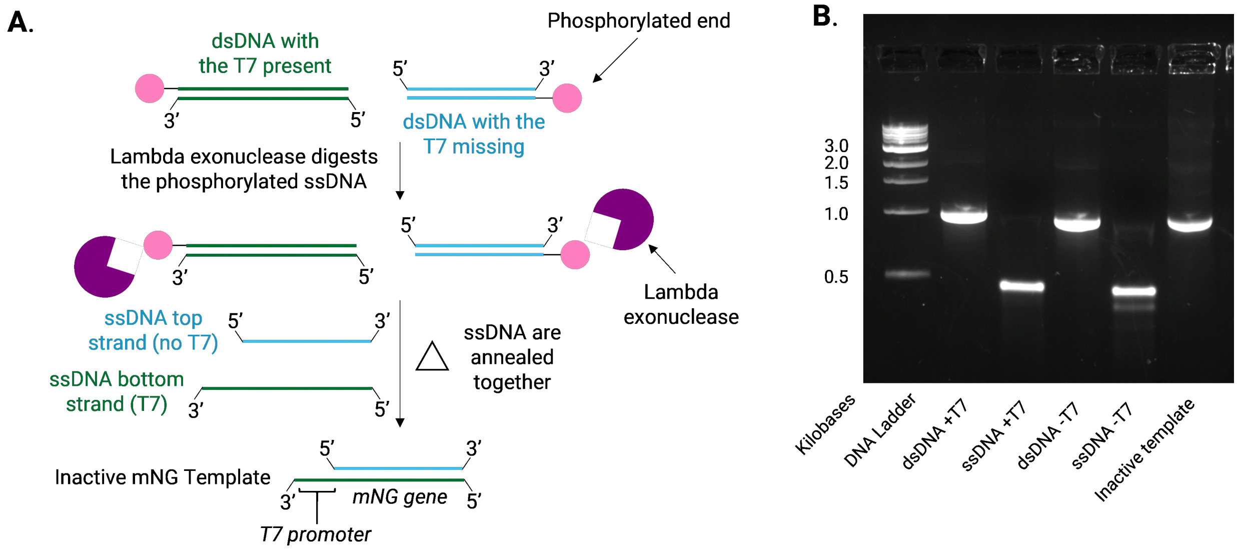

Fig. S6. Synthesis of the inactive mNG template. **(A)** Schematic showing the synthesis of dsDNA with and without the T7 promoter present and with the top and bottom strand, respectively, phosphorylated, and the subsequent digestion of the phosphorylated strands by lambda exonuclease prior to annealing to create the inactive mNG template. Note: promoter (23 base pairs) and gene (~700 base pairs) length are not to scale. **(B)** Native 1.5% (w/v) agarose gel quantifying the synthesis of the inactive template with the dsDNA with (dsDNA +T7) and without (dsDNA -T7) the T7 promoter region present, its digestion to the ssDNA bottom strand with the T7 promoter present (ssDNA +T7) and the ssDNA top strand without the T7 promoter (ssDNA -T7), prior to the annealing and formation of the inactive template (inactive template).

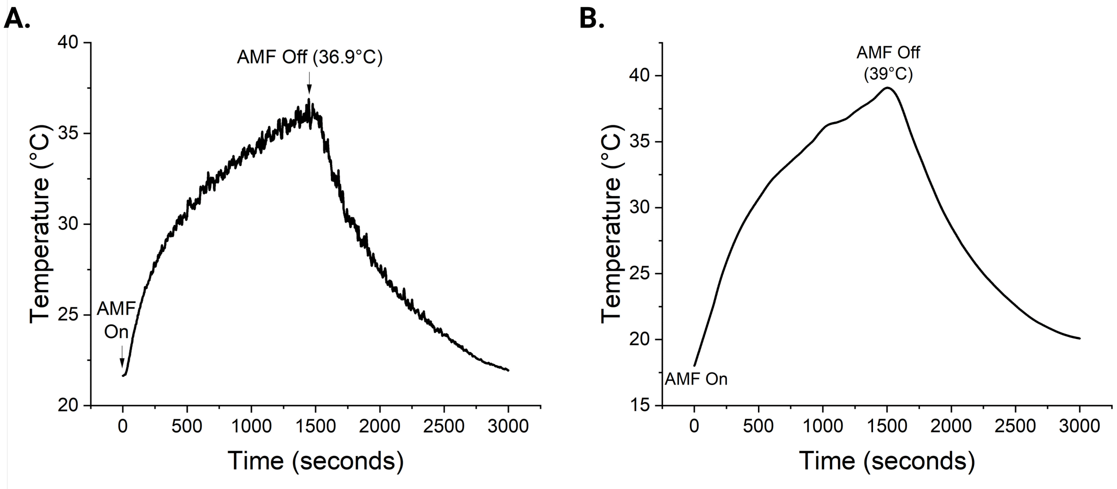

Fig. S7. Fibre optic probe data for the global heating experienced by bulk cell-free protein synthesis reactions in the alternating magnetic field. (A) Fibre optic probe data from the probe being suspended in air inside the cavity of the solenoid coil. **(B)** Fibre optic probe data from the probe being submerged in the bulk CFPS solution and in the presence of magnetically-activated SNAs, inside the solenoid coil and exposure to an AMF for 25 min.

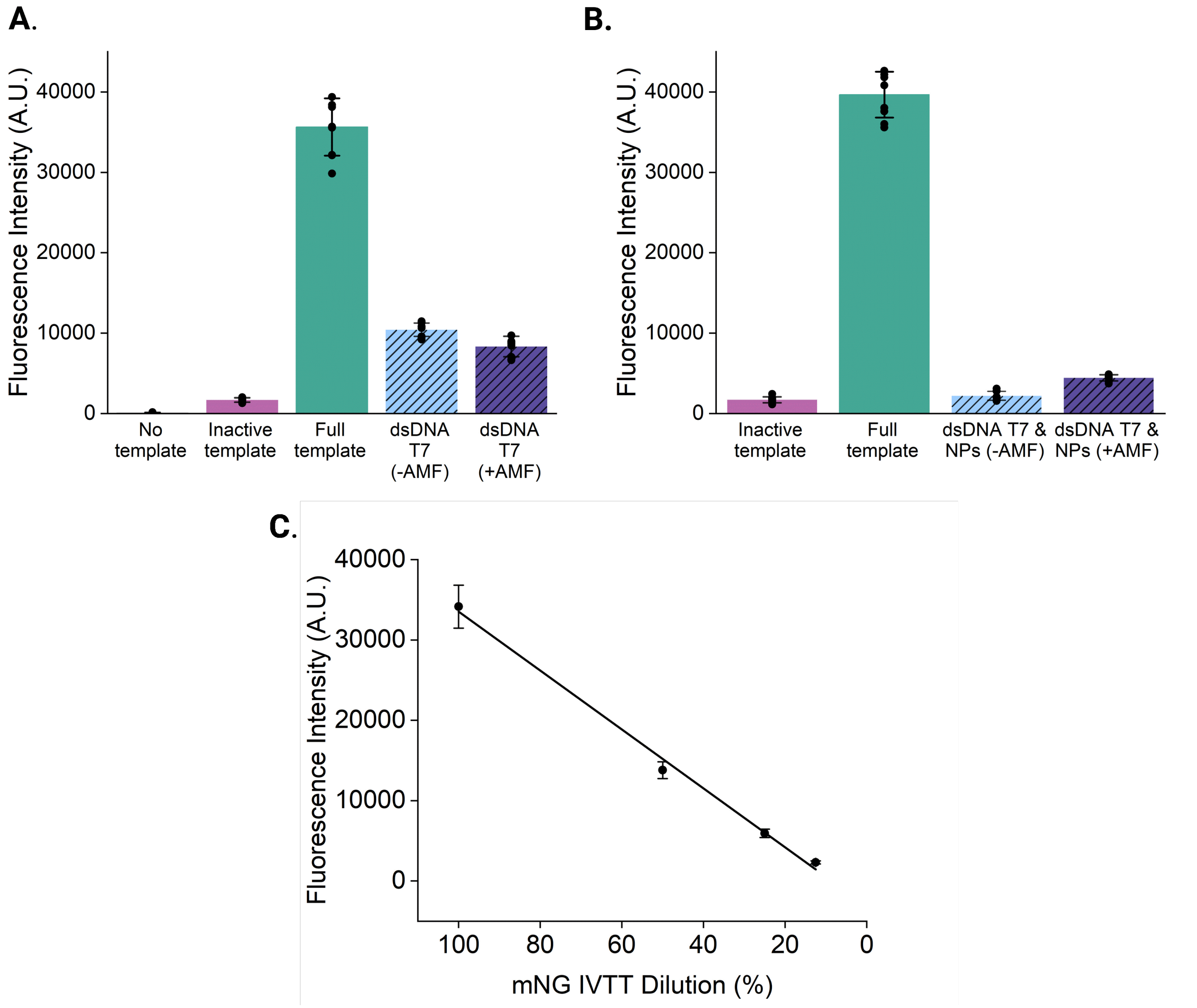

Fig. S8. Cell-free protein synthesis controls with an alternating magnetic field. (A) Cell-free protein synthesis of mNG in the presence of dsT7 promoter with and without an AMF. Datasets are representative of n = 9 technical replicates. Data is presented as mean values +/– SD. **(B)** Cell-free protein synthesis of mNG in the presence of dsT7 promoter and DBCO-modified IONPs@SiO_2 with and without an AMF. Datasets are representative of n = 9 technical replicates. Data is presented as mean values +/– SD. **(C)** Serial dilutions of bulk cell-free protein synthesis, whereby a linear relationship is observed between fluorescence intensity of the mNG protein synthesised and the concentration of the bulk cell-free protein synthesis, diluted with the outer buffer. Datasets are representative of n = 9 technical replicates. Data is presented as mean values +/– SD.

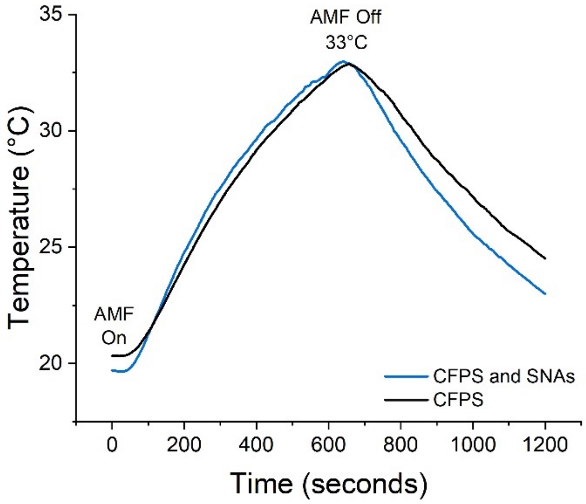

Fig. S9. Fibre optic probe data for the global heating experienced by the synthetic cells in the alternating magnetic field. Fibre optic probe data from the probe being submerged in the CFPS solution and both in the presence and absence of the magnetically-activated SNAs, inside the solenoid coil and exposure to an AMF for 10 min.

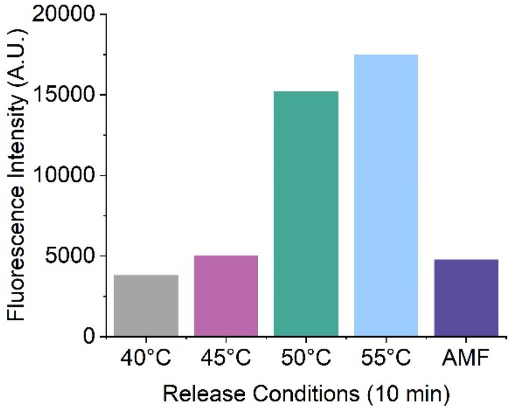

Fig. S10. Release studies of the magnetically-activated SNAs. Release studies and incubation of the magnetically-activated SNAs, T7 promoter bottom strand in solution, and the CFPS buffer (50 mM HEPES, 400 mM potassium glutamate and 200 mM glucose (pH 7.6)) at 40°C, 45°C, 50°C and 55°C, and compared to AMF exposure. Relative concentration of the released T7 promoter top strand was inferred from a PicoGreen assay, measuring the newly-formed dsDNA upon annealing to the T7 promoter bottom strand in solution. The fluorescence intensity of the dsDNA present after 10 min exposure to an AMF (30 mT, 103.4 kHz) was comparative to that after 10 min at 45°C. It can be inferred that the temperature at the surface of the SNA is heated to around 45°C during AMF exposure. Release is higher at 55°C as this is near the melting temperature (Tm) of the duplex.

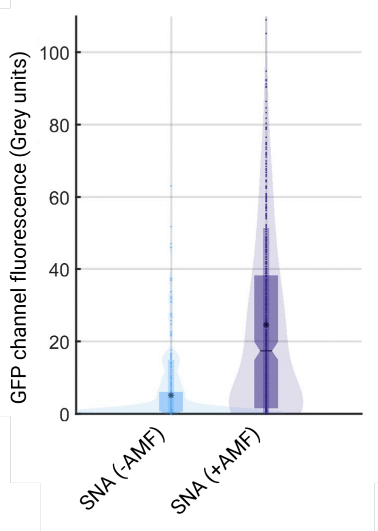

Fig. S11. Quantification of mNG expression in an opaque blocking material. mNG expression in the individual GUVs using a circle detection-based image analysis script. Mean fluorescence intensity (SNA, no AMF) = 5.06 grey units and mean fluorescence intensity (SNA, AMF) = 24.55 grey units. Datasets are representative of n = 2 biological replicates. The box plot, notch and asterisk represent the interquartile range, median and mean fluorescence intensity respectively.

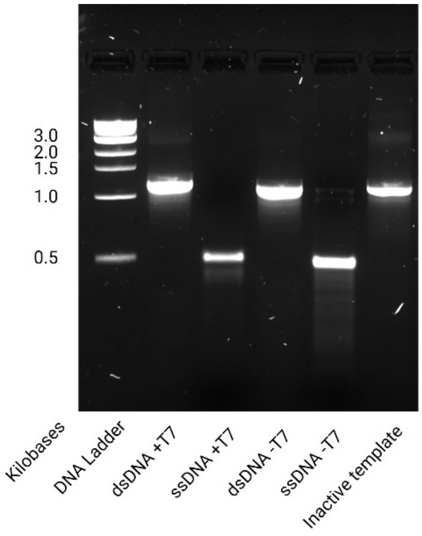

Fig. S12. Synthesis of the inactive α-HL template. Native 1.5% (w/v) agarose gel quantifying the synthesis of the inactive template with the dsDNA that is with (dsDNA +T7) and without (dsDNA -T7) the T7 promoter region present, its digestion to the ssDNA bottom strand with the T7 promoter present (ssDNA +T7) and the ssDNA top strand without the T7 promoter (ssDNA -T7), prior to the annealing and formation of the inactive template (inactive template).

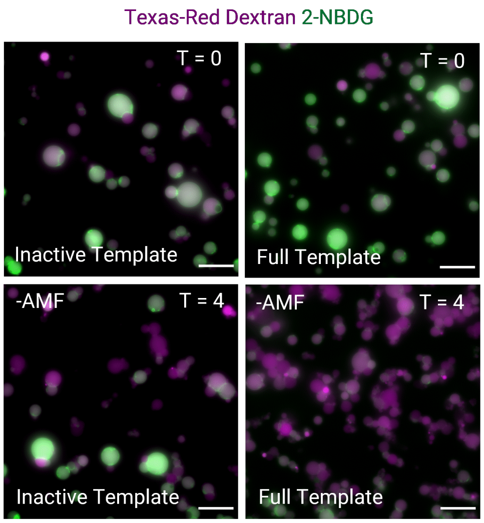

Fig. S13. Alpha-hemolysin expression and small molecule cargo release from synthetic cells. Epifluorescence microscopy images of in-situ α-HL expression in synthetic cells comparing the inactive α-HL template (negative control) to the full α-HL template (positive control), both without exposure to an AMF. Synthetic cells (visualized through the encapsulated Texas-red dextran) that contained only the inactive α-HL template expressed minimal α-HL, visualised by the retention of 2-NBDG fluorescence after 4 h of incubation. Synthetic cells containing the full α-HL template (dsDNA T7 promoter region present) released the 2-NBDG, tracked by the loss of 2-NBSG fluorescence after incubation for 4 h and indicative of expression of α-HL. Images are representative of n = 4 independent experiments. Scale bar = 20 μm.

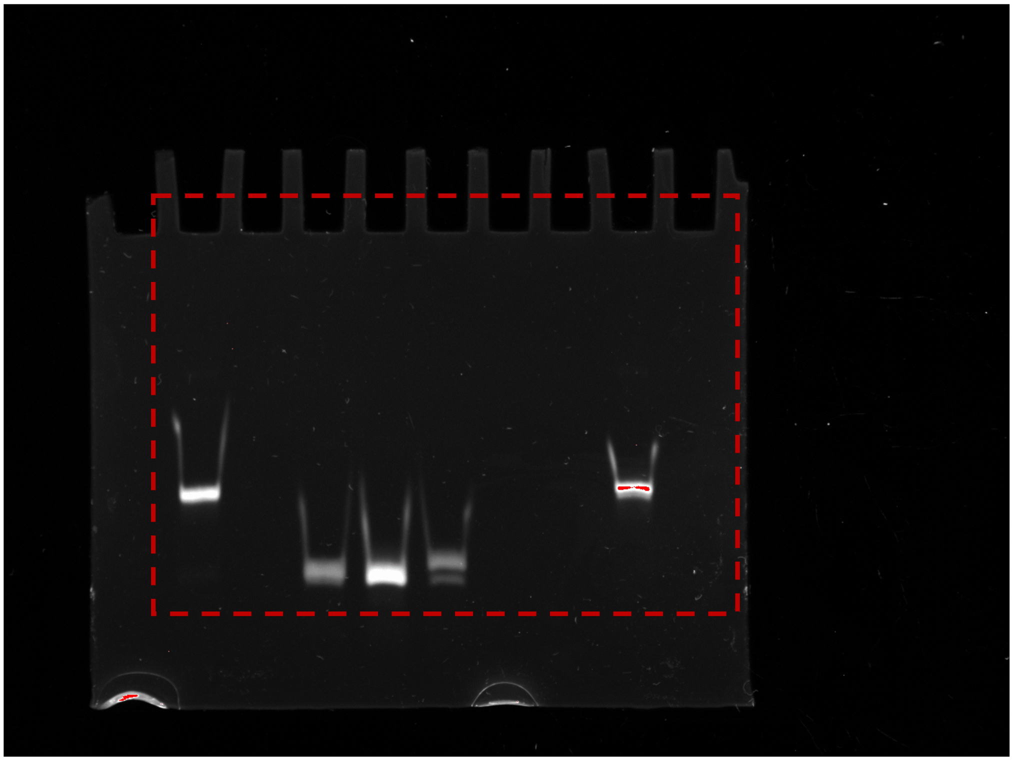

Uncropped gel from supplementary figure S2.

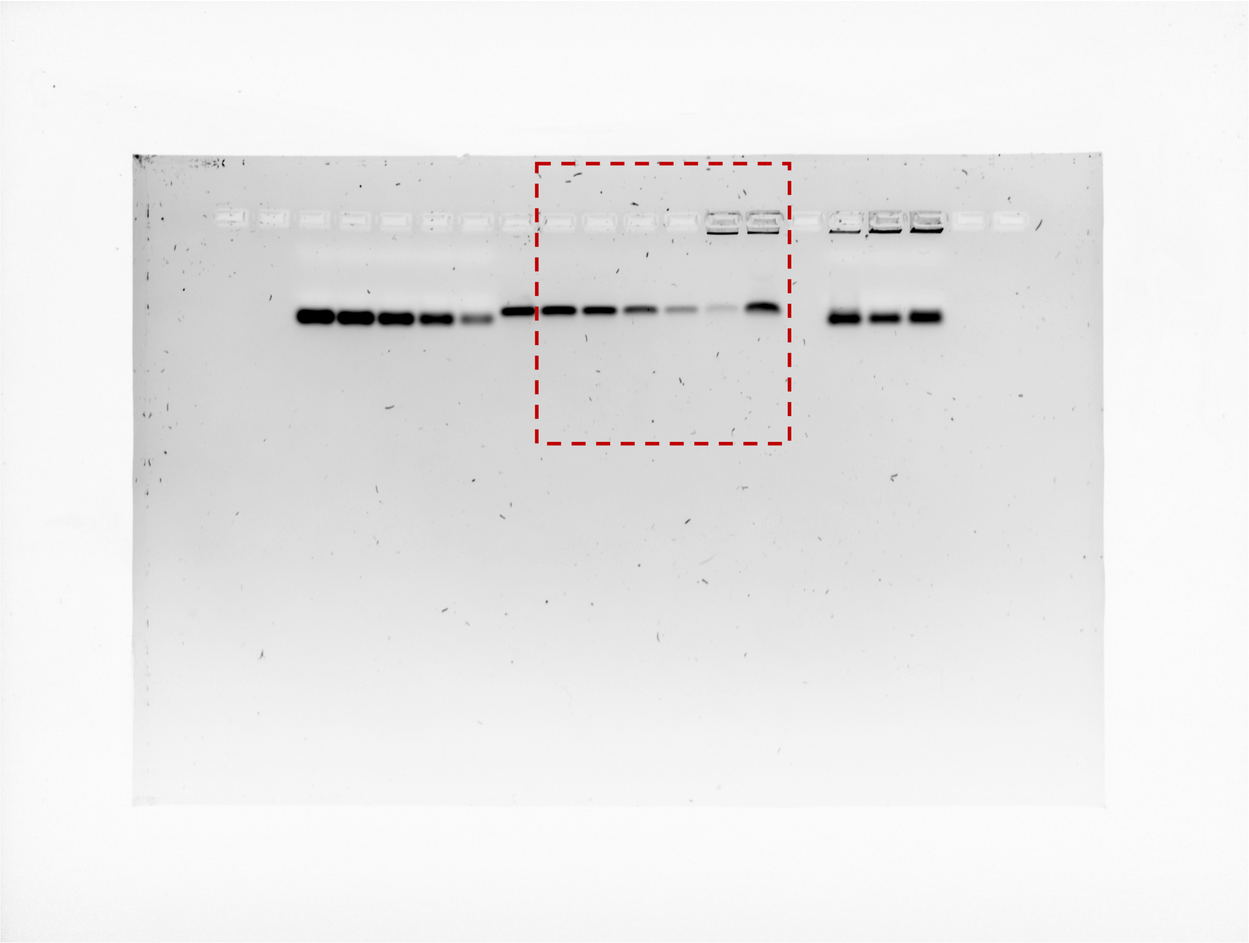

Uncropped gel from supplementary figure S4.

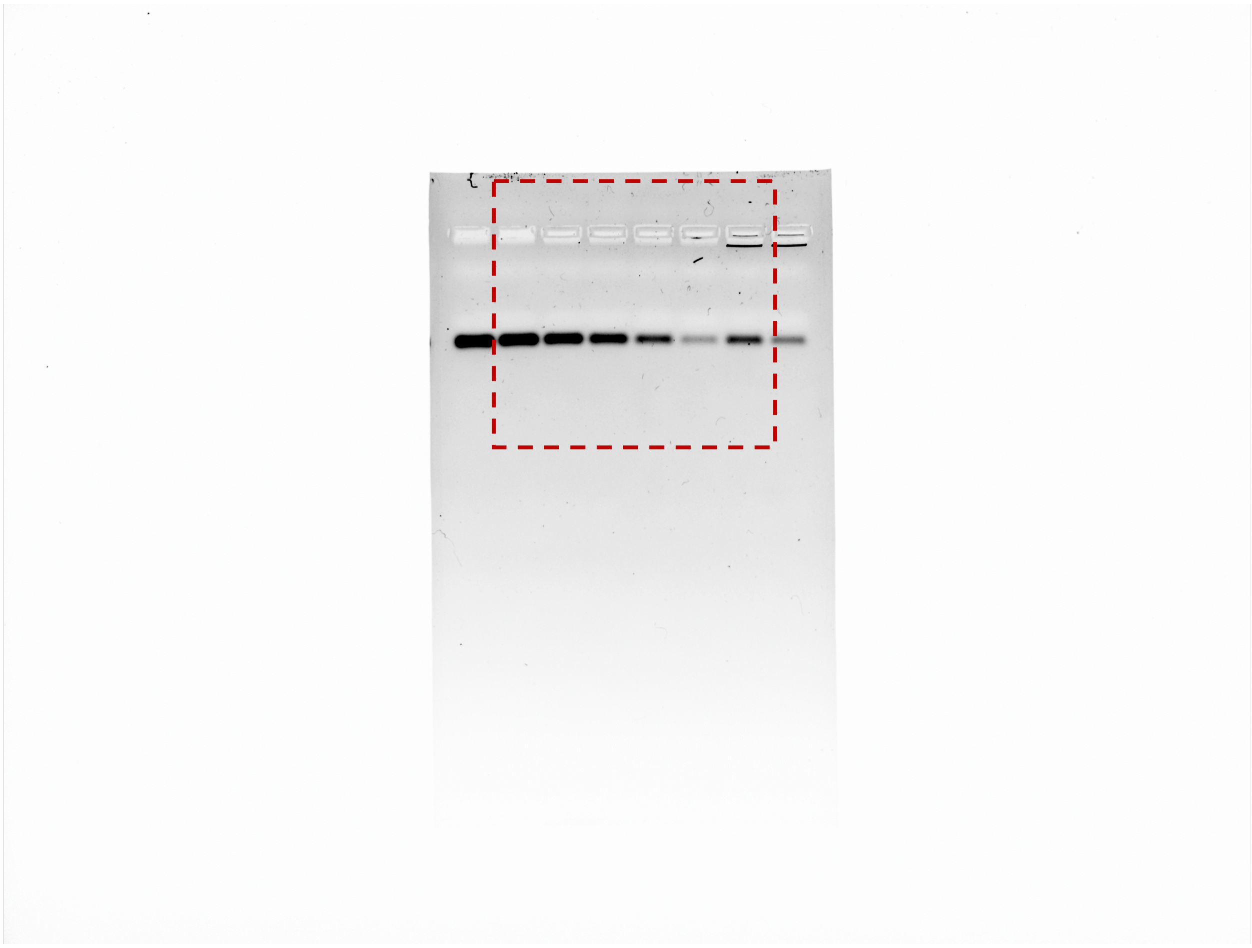

Uncropped gel from supplementary figure S5.

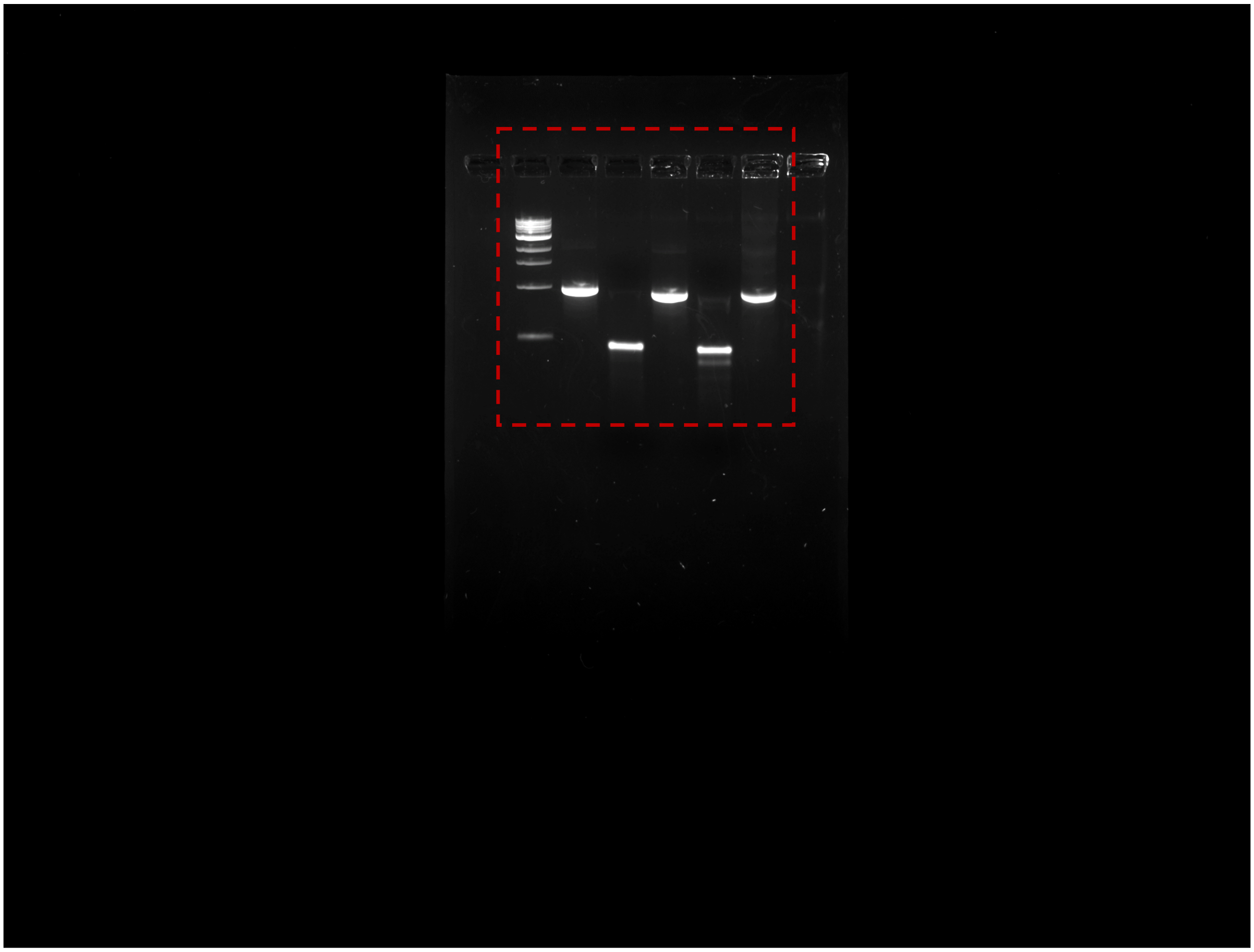

Uncropped gel from supplementary figure S6. DNA Ladder = 1 kb (NEB).

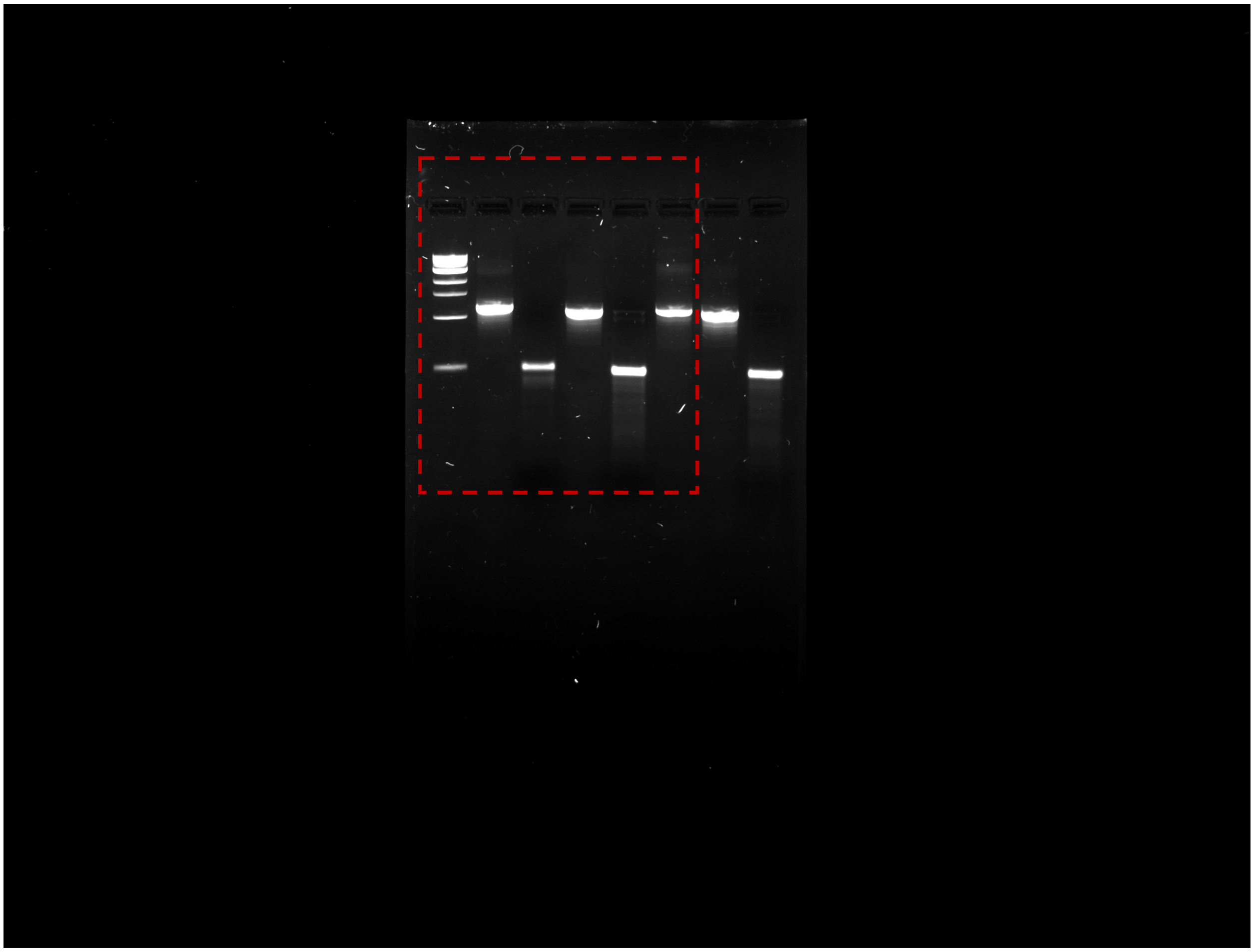

Uncropped gel from supplementary figure S12. DNA Ladder = 1 kb (NEB).

References

1. Nanoparticle Volume, Mass and Concentration. *nanoComposix* https://nanocomposix.com/pages/nanoparticle-volume-mass-and-concentration.
2. Kotagiri, N. *et al.* Antibody Quantum Dot Conjugates Developed via Copper-Free Click Chemistry for Rapid Analysis of Biological Samples Using a Microfluidic Microsphere Array System. *Bioconjug. Chem.* **25**, 1272–1281 (2014).
