# Supplementary figures and images for: Magnetic activation of spherical nucleic acids enables the remote control of synthetic cells
Source: Nat Chem. 2025 Sep 2;17(10):1505–13. doi: 10.1038/s41557-025-01909-6 (PMC12491065; doi:10.1038/s41557-025-01909-6)

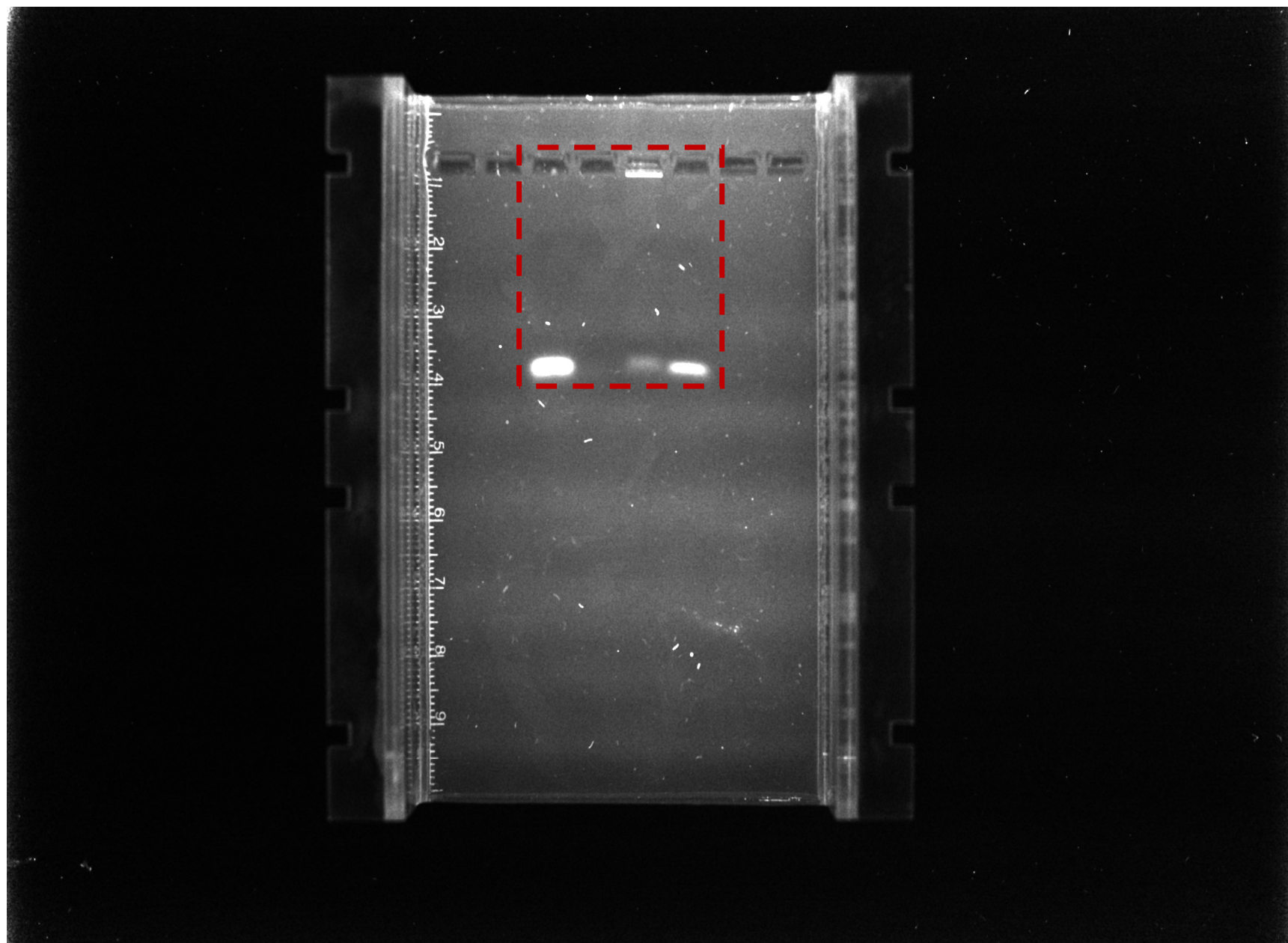

Supplement: Supplementary file 3 — An uncropped image of the GelRed® stained 1.5 (w/v)% TBE agarose gel showing the agarose purification of the magnetically-activated SNAs. [file 41557_2025_1909_MOESM3_ESM.pdf]
